# Yeast culture in weaned lamb feed: a proteomic journey into enhanced rumen health and growth

**DOI:** 10.1186/s40104-025-01223-8

**Published:** 2025-08-01

**Authors:** Xueqiang Li, Xiaolin Yang, Hui Chen, Shixiong Liu, Puguo Hao, Jie Ning, Yingga Wu, Xi Liang, Yufei Zhang, Dacheng Liu

**Affiliations:** 1 College of Veterinary Medicine, Inner Mongolia Agricultural University, Hohhot, 010018 China; 2 Key Laboratory of Clinical Diagnosis and Treatment of Animal Diseases, Ministry of Agriculture, Hohhot, 010018 China; 3 National Center of Technology Innovation for Dairy, Hohhot, 010018 China

**Keywords:** Apoptosis, Cell cycle, Proteome, Rumen epithelial, Weaned lamb, Yeast culture

## Abstract

**Background:**

Using yeast culture as additives in ruminant feed prevents rumen microbial dysbiosis, enhances performance, and regulates rumen pH. The yeast culture used in this study was developed in-house, and has been shown to promote rumen epithelial growth in several sheep trials. Changes in protein expression associated with the promotion of rumen epithelial development following the addition of yeast culture, along with the associated molecular mechanisms, remain unknown. We used 20 45-day-old weaned lambs to investigate the specific proteins and molecular mechanisms involved in these processes. Half of the lambs were fed yeast culture, and the other half were used as controls.

**Results:**

Yeast culture enhanced growth performance, facilitated rumen fermentation, and promoted rumen papilla development in weaned lambs. Proteomics data identified 4,831 proteins in the rumen epithelial tissue of lambs, comprising 87 upregulated and 425 downregulated proteins. Administration of yeast culture activated multiple molecular functions within rumen epithelial cells, including oxidative phosphorylation, glutathione metabolism, apoptosis, cell cycle, and vitamin digestion and absorption. The expression of proteins associated with cell cycle regulation increased, whereas those associated with apoptosis decreased. Administration of yeast culture also reduced the duration of the G_0_/G_1_ phase of rumen epithelial cells and accelerated the cell cycle. Furthermore, yeast culture showed increased cyclin D1, cyclin-dependent kinase (CDK)2, CDK4, CDK6, and cyclin E1 expressions and decreased cytochrome C (Cyto-c), Bcl-2-related X protein (Bax), cleaved caspase 3 (C-caspase 3), caspase 3, and cleaved caspase 7 (C-caspase 7) protein expressions. Yeast culture upregulated the insulin-like growth factor-1 receptor (*IGF-1R*) and insulin-like growth factor-binding protein 5 (*IGFBP-5*) mRNA expressions in rumen epithelial cells.

**Conclusions:**

Yeast culture facilitates rumen epithelial development by regulating the cell cycle and IGF-1 signaling and reducing the expression of proteins associated with apoptosis in rumen epithelial cells. The findings of this study provide novel insights into the molecular mechanisms through which yeast culture promotes rumen epithelial development in weaned lambs.

**Supplementary Information:**

The online version contains supplementary material available at 10.1186/s40104-025-01223-8.

## Background

Sheep farming in China is shifting from traditional grazing methods or supplemental grazing to more intensive indoor practices. To enhance productivity, the natural weaning age of young animals is frequently expedited in indoor settings feeding. However, their digestive and immune systems remain immature. Rumen development at a young age is critical for sheep. Growth and development of the rumen epithelium directly affects ruminant performance and health. Underdeveloped rumen can result in indigestion, stunted growth, weakened immunity, or severe outcomes such as diarrhea and higher mortality rates [1, 2].

Improved feeding management and nutrition can facilitate optimal growth, feed utilization, and health of young calves [3]. However, research into the mechanisms through which different dietary components promote the growth and health of animals is ongoing in the field of animal nutrition. Microecological agents have demonstrated considerable potential for promoting growth [4–9], enhancing the immune function of animals, reducing oxidative stress, improving disease resistance, and promoting floral balance and nutrient digestibility in the gastrointestinal tract.

Yeast cultures have been shown to increase animal productivity, improve immunity, prevent diarrhea, enhance antioxidant capacity, and modulate inflammatory factors, among other beneficial effects [10, 11].

Incorporating yeast into domestic animal diets has been a common practice. Including yeast cultures in the diet of pigs, cattle, horses, and sheep enhances production performance [3, 12, 13].

Yeast is a widely used component of ruminant production that enhances feed efficiency by competing with other microorganisms within the rumen and prevents rumen acidosis through its fermentative activity [11, 14]. The incorporation of yeast cultures into beef cattle diets enhances their growth and development and optimizes their nutritional digestibility and average daily weight gain [15]. The incorporation of yeast cultures into total mixed diets has been demonstrated to enhance the growth performance of lambs, primarily due to enhanced digestion, absorption, and utilization of fiber [16]. Furthermore, yeast cultures effectively restore intestinal microbial balance in digestive disorders [17] as they contain various metabolites, including proteins, yeast cell wall polysaccharides, lipids, vitamins, peptides, amino acids, nucleotides, organic acids, compounds, and antioxidants, in addition to live and dead yeast cells [16]. These metabolites can increase the feed intake of ruminants, maintain ruminal environment, increase feed utilization, and enhance the immunity of the body, thereby improving animal performance. Incorporating yeast culture products into animal husbandry has improved productivity, advanced green farming strategies, alleviated the global food crisis, and made substantial contributions to the advancement of both human and livestock industries. Several probiotic-fermented products for ruminants have been developed; however, their quality and usage greatly vary.

This research utilized a yeast culture, a microecological preparation created by our team specifically for ruminants. It comprises a limited number of live yeast cells along with a certain quantity of metabolites.

The two dominant yeast strains have significant advantages in terms of cell biomass, enzyme production ability, and ability to secrete active substances. Using these two yeast strains, YC was produced in a specific culture medium using special fermentation processes [18]. In a preliminary study, the rumen epithelium of sheep supplemented with yeast culture showed significantly better development, with longer rumen papillae and thicker rumen walls [19].

The rumen plays a pivotal role in the digestive process of ruminants by facilitating the breakdown of high-fiber feed through microbial fermentation. In particular, the rumen epithelium of young ruminants must quickly adapt to solid feed to enhance nutrient absorption and transport and improve immune function [20, 21]. Therefore, it is important to implement nutritional interventions to enhance early rumen development to reduce weaning stress. Supplementation with yeast cultures during the pre-weaning period is effective in increasing rumen weight and nipple size and enhancing the rumen barrier to harmful substances. Ultimately, this has a positive overall impact on the health and growth of young ruminants [22–25]. The precise molecular mechanisms through which yeast culture facilitates rumen papillary growth remain unclear. High-energy diets facilitate the expansion of the rumen epithelium in goats, predominantly due to the acceleration of the cell cycle [22, 26, 27]. Furthermore, butyrate infusion stimulates rumen mucosal development in calves, primarily by reducing rumen epithelial cell apoptosis [28–31]. Therefore, it can be postulated that yeast culture facilitates the development of the rumen epithelium in lambs by accelerating the cell cycle and inhibiting apoptosis.

Despite the extensive literature on yeast culture in ruminants, data regarding the underlying mechanisms remain lacking. The potential of yeast cultures to enhance rumen fermentation and growth performance has been re-examined in light of global regulations prohibiting the use of antibiotics in livestock feed. However, the efficacy of yeast cultures in promoting rumen epithelial development in lambs, particularly in terms of the underlying molecular mechanisms, has been overlooked.

Therefore, the objective of this study is to examine the impact of yeast culture on the growth performance, rumen fermentation, and rumen epithelial development of weaned lambs. To this end, proteomics technology was employed to conduct a comprehensive and systematic investigation of the molecular mechanisms through which yeast culture facilitates rumen papillary growth in lambs.

## Methods

### Yeast culture preparation

**Fig. 1:**
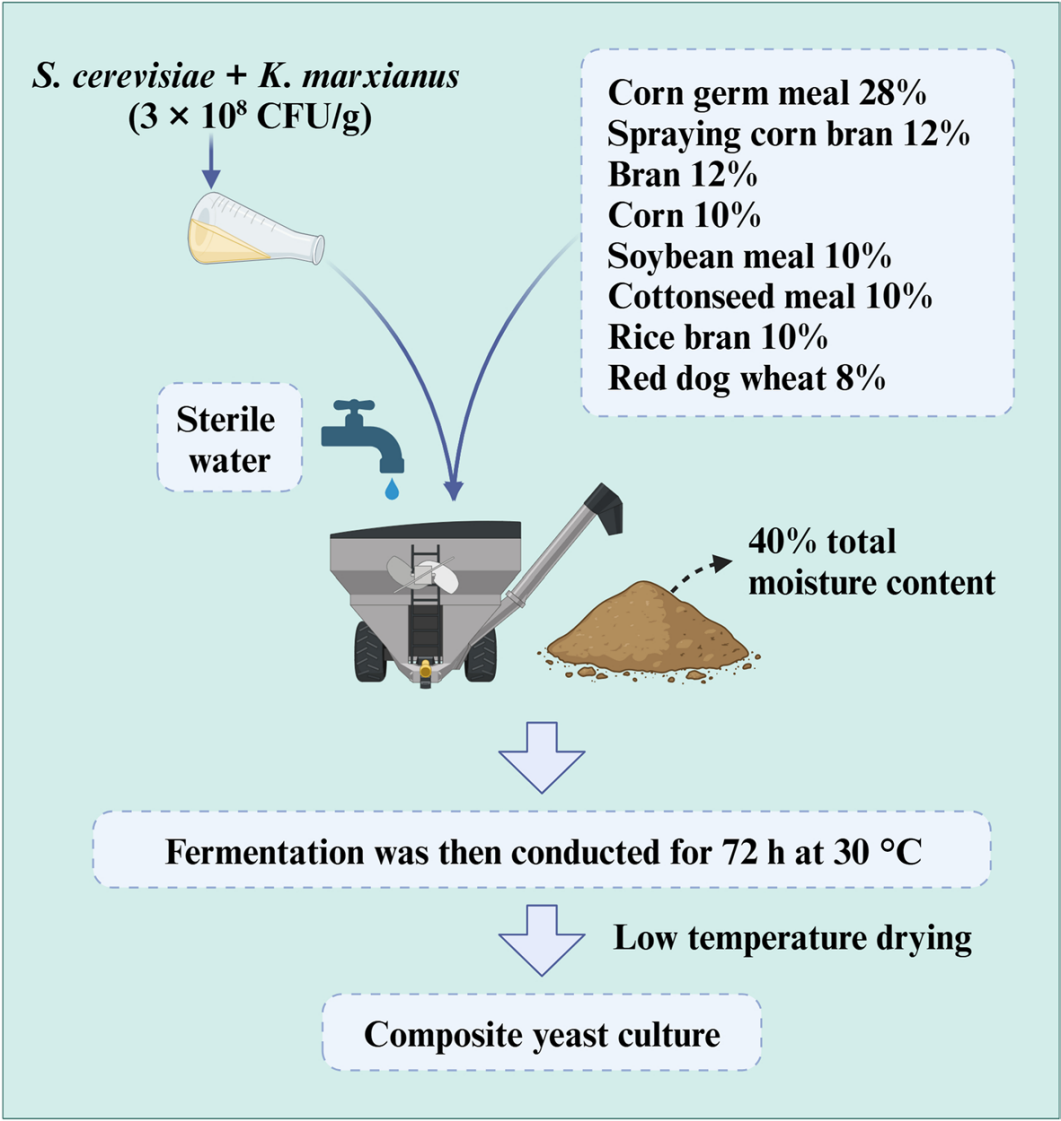
Flowchart for the preparation of composite yeast culture

*Saccharomyces cerevisiae* and *Kluyveromyces marxianus* strains with favorable fermentation traits were isolated from naturally fermented horse milk. A pilot production was performed at Kehong Feed Co., Ltd. in Inner Mongolia, China, using a base matrix consisting of various ingredients (Fig. 1). A 1:1 mixture of the yeast strains *S. cerevisiae* and *K. marxianus* was inoculated at a concentration of 10% per 1,000 kg of a wet-mixed matrix with sterile water, resulting in a total moisture content of 40%. Fermentation was conducted aerobically for 72 h at 30 °C. The nutrient composition is presented in Table 1.

**Table 1:** Nutrient composition of mixed feed and yeast culture (feed-based)

Item	Mixed feed	Composite yeast culture
Dry matter, %	91.95	92.23
Crude protein, %	19.51	20.21
Neutral detergent fiber, %	34.06	33.89
Acid detergent fiber, %	19.98	19.91
Live yeast cells, CFU/g		7.2 × 10^6^
Lactic acid, mmol/kg		382.52

### Animals, diets, and experimental design

The study was conducted at Inner Mongolia Fuchuan Breeding Technology Co. Ltd. (Bayannur, Inner Mongolia, China), where 20 45-day-old healthy male lambs (Dorper × Thin-tailed Han) from the same batch with similar weight (22.56 ± 1.66 kg), were randomly assigned to either a control or test group (*n* = 10 lambs/group). Each lamb was housed in an individual pen measuring 1.5 m × 1.8 m. The control group (NC) received a basal diet, whereas the experimental group (NM) received daily supplementation with 30 g of yeast culture added to the basal diet. The experimental period lasted 37 d, including a pre-feeding period of 7 d and a formal test period of 30 d. At the end of the experiment, six lambs were randomly selected from each group for slaughter and sampling. Feeding was performed at 7:30 and 17:30 daily, with free access to feed and water.

The basal diet for the lambs was formulated according to the Nutrient requirements of meat-type sheep and goat (NY/T 816–2021) [32]. The composition and nutritional levels of the diets are listed in Table 2. Samples were collected weekly for measurement and analysis. The dry matter content was determined using the oven-drying method (DZF-6210, YIHENG, Shanghai, China) as outlined in GB/T 6435–2014 [33], whereas the crude protein content was determined using a fully automated Kjeldahl nitrogen determination method (NKD6280, WANGHAI, Shanghai, China) as specified in GB/T 6432–2018 [34].

**Table 2:** Dietary composition and nutrient levels of experimental diets (%, DM basis)

Ingredients	NC	NM
Corn grain	29.00	29.00
Alfalfa meal	10.00	10.00
Corn stalk	9.50	9.50
Peanut vine	9.30	9.30
Germ meal	8.50	8.50
Sunflower cakes	8.00	8.00
Soybean meal	6.00	6.00
Cotton meal	6.00	6.00
Distillers dried grains with solubles	5.50	5.50
Sunflower seed skin	4.00	4.00
NaCl	0.50	0.50
Limestone	0.50	0.50
CaHPO_4_	0.50	0.50
Premix^a^	2.70	2.70
Total	100.00	100.00
Nutrient levels^b^		
Dry matter	88.72	88.67
Metabolizable energy, MJ/kg	10.12	10.09
Crude protein	15.75	15.82
Neutral detergent fiber	33.28	33.31
Acid detergent fiber	21.49	21.42

*NC* Basal diet control group, *NM* Basal diet + composite yeast culture

^a^Premix provided the following per kilogram of diets: vitamin A 3,500 IU, vitamin E 20 IU, vitamin D 1,200 IU, Fe 60 mg, Mn 45 mg, Zn 60 mg, Cu 12 mg, Se, 0.2 mg, I 0.6 mg, nicotinic acid 60 mg, Co 20 mg, Ca 2 g, P 1 g, NaCl 5 g

^b^Metabolizable energy was calculated as described, and the others were measured values

The neutral detergent fiber (GB/T 20806–2022) [35] and acid detergent fiber (NY/T 1459–2022) [36] contents were determined using F2000 fiber analyzer (Hanon, Jinan, China).

The study protocol was approved by the Institutional Animal Protection and Utilization Committee of Inner Mongolia Agricultural University (Approval No. NND2022104). All procedures adhered to the National Research Council’s guidelines (2022–6–10/SYXK 2022–0031) for animal welfare, including humane handling, minimization of distress, and compliance with the 3R principles (Replacement, Reduction, Refinement).

### Dietary intake and growth

Throughout the experiment, weights were measured before the morning feeding at the commencement and conclusion of the experiment, and the average daily weight gain was calculated. Animal health was assessed daily by licensed veterinarians through visual inspections, behavioral observations (activity levels, social interactions), fecal analysis, and appetite tracking. Abnormal cases underwent comprehensive vital sign monitoring (body temperature). Feeds were provided, and the resulting residue was collected and weighed daily, followed by an analysis of dry matter intake (DMI).

### Preparation of plasma samples

Blood samples were collected from the jugular vein using 5-mL anticoagulant tubes on the morning of days 1 and 30, prior to feeding. All samples were centrifuged at 3,000 r/min for 10 min to isolate plasma, ensuring consistency with fasting conditions and minimizing dietary interference. The plasma was dispensed into 2-mL sterile and enzyme-free cryopreservation tubes, numbered and recorded, and stored at –80 °C.

### Morphometric and histomorphometric analyses

A 1 cm^2^ sample of rumen tissue from the rumen ventral sac was fixed in 4% neutral paraformaldehyde solution for histomorphometric microscopy analysis. Briefly, rumen epithelial tissues were collected and prepared as paraffin-embedded sections, followed by dehydration with varying alcohol concentrations (100%, 75%, 50%, and 25%). Histological alterations in rumen epithelial tissues were visualized using hematoxylin and eosin (H&E) staining with AxioScan.Z1 slide scanner (Zeiss, Oberkochen, Germany) [37]. Each lamb had one slide with two images for a total of 20 replicates per measurement. The thickness of the ruminal epithelial layers was measured using a 40× objective lens. The microscopist was blinded to the treatment group during the analysis.

### Determination of ruminal pH and volatile fatty acid concentrations

Four hours post-morning feeding, animals were euthanized, and the rumen was immediately isolated and incised. The contents were homogenized with a sterile stainless-steel spatula and filtered through four layers of sterile gauze [38]. The filtered rumen fluid was analyzed for pH within 10 min using a calibrated pH meter (PB-10, Sartorius, Germany). For VFA quantification, 10 mL of rumen fluid was stabilized with 25% (w/v) metaphosphoric acid and stored at −80 °C until analysis. The concentration of VFAs in the rumen fluid was determined using gas chromatography (GC-14B, Shimadzu, Japan) under the following conditions: column temperature of 130 °C, vapor chamber temperature of 180 °C, and detector temperature of 180 °C. The carrier gases used were nitrogen (60 kPa), hydrogen (50 kPa), and oxygen (50 kPa).

### Sample preparation and trypsin digestion of label-free proteomes

Ruminal epithelial tissue was collected from the ventral sac within 5 min post-slaughter. The muscular and serosal layers were removed through blunt dissection, and the isolated epithelium was rinsed with sterile PBS [38]. The rumen epithelial tissue samples were immediately flash-frozen in liquid nitrogen (−196 °C) to arrest metabolic activity and then transferred to a –80 °C freezer for long-term preservation, ensuring protein integrity for subsequent proteomic profiling.

Protein samples were prepared as previously described [39]. Five samples per group were selected for proteomic analysis, ground in liquid nitrogen, lysed with PASP lysis buffer, and quantified using the Bradford assay. A 20-μg protein sample was analyzed using sodium dodecyl sulfate–polyacrylamide gel electrophoresis (SDS-PAGE) gel electrophoresis. Proteins were dissolved in Tris-HCl with urea, incubated at 60 °C, alkylated with iodoacetamide, and incubated at room temperature. Proteins on the membrane were dissolved in ammonium bicarbonate (NH_4_HCO_3_). The digested protein was desalted using a C18 column and freeze-dried prior to analysis.

### Liquid chromatography with tandem mass spectrometry (LC–MS/MS) analysis

LC–MS/MS analysis was conducted as previously described [40]. Mobile phases A and B were prepared, and the lyophilized powder was dissolved in solution A. Following centrifugation, 1 μg of the supernatant was injected into a C18 Nano-Trap column. Peptides were eluted using a gradient of 2%–95% buffer B for 2 h and analyzed using tandem mass spectrometry (Thermo Fisher, Germany) for data-dependent acquisition. The MS analysis parameters included an electrospray voltage of 2.0 kV, precursor scan range of 350–1500 *m/z* at a resolution of 60,000 in Orbitrap, MS/MS fragment scan of > 100 *m/z* at a resolution of 15,000 in HCD mode, normalized collision energy setting of 30%, dynamic exclusion time of 30 s, AGC for the full MS target and MS^2^ target of 3 × 10^6^ and 1 × 10^5^, respectively, and the 20 most abundant precursor ions above a threshold ion count of 10,000 following one MS scan [22].

### Data analysis and bioinformatics analyses

The DIA data for this experiment were analyzed using the DIA-NN (v 1.8) search engine with default parameters. The Ovis_aries_9940_PR_20230322 database was used in the present study. FASTA contains 23,110 sequences. The digestion mode was trypsin/P, with a maximum of one missed cut allowed. Fixation modifications included short-term M excision and carbamidomethylation. Deep-learning algorithms were employed to generate theoretical spectral libraries, and inverse libraries were incorporated to assess the false-positive rate (FDR) resulting from random matching. FDR for precursor identification was 1%. Additional data filtering was required to ensure the production of high-quality analysis outcomes. The filtering criteria for the identification results included setting FDR for the precursor and protein at 1% and requiring that the identified proteins contain at least one unique peptide. The automated software IQuant was used for protein quantification, and downstream analysis was conducted on proteins with FDR of < 1%. Significant upregulation was defined as a change in differential expression exceeding 1.5 with a *P*-value < 0.05, whereas significant downregulation was defined as a change of < 1.5.

In this study, the OmicsBean tool was used to analyze multi-omics data [41], specifically focusing on the Kyoto Encyclopedia of Genes and Genomes (KEGG) pathway, principal component analysis (PCA), and hierarchical clustering analysis of all proteins expressed. In addition, the Gene Ontology (GO) enrichment analysis of differentially expressed proteins (DEPs) was conducted using DAVID (Version 6.8) [21].

Furthermore, protein–protein interaction (PPI) network analysis of DEPs was conducted using the Search Tool for the Retrieval of Interacting Genes (STRING) database (version 11.0) and visualized using Cytoscape (Version 3.7.2) [42].

### Flow cytometry

The rumen epithelial tissue (10 g wet weight), obtained from the rumen ventral blind sac, was digested with 0.25% trypsin and treated with 0.02% ethylenediaminetetraacetic acid (EDTA) in D-Hank’s solution. The harvested cells were rinsed thrice with cold PBS and preserved in 70% ethyl alcohol at 4 °C overnight. Subsequently, intracellular DNA was stained with propidium iodide (50 μg/mL) at 4 °C in the dark. The resulting samples were analyzed using a flow cytometer (FACSVantage SE, BD Biosciences, America) to identify variations in the cell cycle distribution. The results were analyzed using ModFit software.

### Immunohistochemistry

The rumen epithelial tissue was fixed in 4% paraformaldehyde (in phosphate-buffered saline [PBS]) for 24 h, dehydrated, and subsequently embedded in paraffin. Following deparaffinization and rehydration, the tissue sections were microwaved in 0.01 mol/L sodium citrate for 15 min. To inhibit enzymatic activity, the rumen epithelial tissue was exposed to 3% H_2_O_2_ for 10 min, followed by two 5-min washes with PBS. Subsequently, the sections were rinsed three times with PBS and incubated in a PBS buffer solution containing 0.25% Triton-X and 1% bovine serum albumin (BSA) for 20 min at room temperature.

The blocking serum was removed, and anti-PCNA antibody (1:100 dilution, Affinity Biosciences, Jiangsu, China) was added and incubated at 4 °C overnight. After rinsing with PBS, the sections were incubated with a goat anti-rabbit secondary antibody (1:500; Boster, Wuhan, China) for 1 h at room temperature. Subsequently, the sections were treated with liquid 3,3′-diaminobenzidine tetrahydrochloride substrate (Boster) and counterstained with Mayer’s hematoxylin (Boster). Finally, slides were dehydrated, mounted, and examined under a microscope. The authors who conducted the study were blinded to animal grouping. Slices were systematically photographed in a non-overlapping manner, proceeding from left to right and top to bottom. Each picture was assigned a number, and random fields were selected using a computer-generated random number method. The final value for each slice was determined by calculating the mean value of a minimum of six randomly selected high-power fields. Quantitative analysis was performed using the Image-Pro Plus 6.0 software (Media Cybernetics, Rockville, MD, USA), and the integrated optical density was computed for statistical analysis [43].

### RNA isolation, cDNA synthesis, and real-time polymerase chain reaction (PCR)

Total RNA was extracted from rumen epithelial tissue using an Axygen RNA kit (Axygen Scientific, Shanghai, China) according to the manufacturer’s instructions. Subsequently, RNA was reverse-transcribed into cDNA using the PrimeScript RT kit (Vazyme, Nanjing, China). The mRNA levels of various genes including cyclin-dependent kinase (*CDK*)1, *CDK2*, *CDK4*, *CDK6*, cyclin A, cyclin A2, cyclin B1, cyclin D1, cyclin E1, *Bax*, *Bad*, *Bcl2*, *caspase 3*, *caspase 8*, cytochrome complex (*Cyt-c*), *Fas*, *TNFR1*, *IGF1*, *IGF1R*, *IGFBP2*, *IGFBP3*, *IGFBP5*, *IGFBP6*, *SLC16A1*, *DRA*, *PATL1*, *HMGCS1*, *HMGCL*, *BDH1* and *BDH2* were quantified using the SYBR Green Master Mix Kit (Roche Applied Science, Mannheim, Germany). Normalization of gene expression was performed against Actin mRNA levels, and data analysis was conducted following the 2^−△△Ct^ method [3]. All primers used for these analyses are listed in Table 3.

**Table 3:** Primer sequences used for real-time PCR

Gene	Primer sequence (5′→3′)	Annealing temperature, °C	Product size, bp
β-Actin	F: GCGGCATTCACGAAACTACC	60	129
	R: TCTGCATCCTGTCTGCGATG		
*CDK1*	F: CGCTTGGAGTTAGGGAGAGC	60	189
	R: CATGGCTACCACTTGACCTGT		
*CDK2*	F: GACTTCGGACTAGCCAGAGC	60	164
	R: CGGGTCACCATCTCAGCAAA		
*CDK4*	F: CAGTGTACAAGGCCCGTGAT	60	176
	R: GACGTCCATGAGCCTGACAA		
*CDK6*	F: TACCTCAGTGGTTGTCACGC	60	134
	R: GGAGTCCAATCACGTTTCTACG		
Cyclin A	F: GGAGATCCTGGACTGGGGT	60	156
	R: TAGAGCGCTGGAATGAAGCC		
Cyclin A2	F: GAACGTCAACCCCGAGAAGT	60	132
	R: TTAAGAGGTGCAACCCGTCG		
Cyclin B1	F: TTGGCTCTGCCTGGATTATTT	60	194
	R: GGCAGATAGCTGGTGAACGA		
Cyclin D1	F: GGCGGGGAACAGTTTCCTAA	60	105
	R: CCCACTTCCGCTAACACCAT		
Cyclin E1	F: TCCTCCAAAGTTGCACCAGT	60	93
	R: GCAACCTTCATGATGATTAATTCCA		
*Bax*	F: GCCCTTTTGCTTCAGGGTTT	60	121
	R: TCAGACACTCGCTCAGCTTC		
*Bad*	F: TTGGGCCCAGAGCATGT	60	126
	R: CAGTGCTTGCTGAGACCTGG		
*Bcl2*	F: GGTCGCATTGTGGCCTTTTT	60	187
	R: CTGCGTTGTTCCCGTAGAGT		
*Caspase 3*	F: ATAGCGCAGGGAACTACAGTC	60	112
	R: GTTTCCGGTGCATAGCAAGTG		
*Caspase 8*	F: TGTCGTCGAGAGTTTGGTGG	60	153
	R: GGCGAATCCTGGAACCTTCA		
*Cyt-C*	F: TTTGTTCAGAAGTGTGCCCAGTG	60	192
	R: CCTGACCTGTCTTTCGTCCAAAC		
*Fas*	F: GCACCACGTGTGAACATGGA	60	204
	R: CACTTGAGGCTGCAGAGACG		
*TNFR1*	F: TGTCCCAACGGCACAGTGAA	60	181
	R: TGCCTGGGTCCTGAGAATCTTT		
*IGF1*	F: CAGTCACATCCTCCTCGCATC	60	254
	R: ACAGTACATCTCCAGCCTCCTCA		
*IGF1R*	F: AACCTGCGGAATATCACCCG	60	198
	R: GATGGTCGTCTTCTCGCACA		
*IGFBP2*	F: GTCGGAAGCCCCTCAAGTTC	60	103
	R: CCAAGGTGATGTTTGCCACC		
*IGFBP3*	F: AGGAAATGGCAGTGAGTCGG	60	100
	R: GAGGTGGGATTTGGAGTCGG		
*IGFBP5*	F: GTTTGCCTGAACGAAAAGAGC	60	105
	R: TGAGTAGGTCTCCTCTGCCATCT		
*IGFBP6*	F: GCCCTCGGGAGAGAATCCTAA	60	146
	R: TCTCAGTGTCTTGTACGCCC		
*SLC16A1*	F: TGGACATAAGCCTATTCAAGCAC	60	98
	R: GCTAAGAAAGACCAGTGGTGTAAAT		
*DRA*	F: AACAACACCCCGAACACCAAT	60	212
	R: AACTTGCGGAAAAGGTGGTCA		
*PATL1*	F: GGCAGCAACAGAATAGAAATCAGC	60	270
	R: GTTCCTTTCTTGGGCCGTCA		
*HMGCS1*	F: CGATGGTGTAGACGCTGGAAAG	60	280
	R: GCCTCCATAGCACGCATTAGTT		
*HMGCL*	F: CAGCGGTTTGACGAAATCTTGA	60	131
	R: TTCTTGGAGACCTCAGCGACTT		
*BDH1*	F: CAGCAGAGGTCATCCGTTCA	60	142
	R: CAGAGGTTCACTTCCGCCAC		
*BDH2*	F: GAGAAGGTGCCAAAGTCATAGCC	60	196
	R: CAGTCCAGGATGGTTCCGTGA		

*CDKs *Cyclin-dependent kinases, *Bax *Bcl-2-associated X protein, *Bad *Bcl-2 asociated death promoter, *Bcl-2 *B-cell lymphoma-2, *Caspase 3 *Cysteinyl aspartate specific proteinase 3, *Caspase 8 *Cysteinyl aspartate specific proteinase 8, *Cyt-C *Cytochrome Complex, *Fas *Factor-related Apoptosis, *TNFR1 *Tumor necrosis factor receptor 1, *IGF1 *Insulin-like growth factor 1, *IGF1R *Insulin-like growth factor 1 receptor, *IGFBPs *Insulin-like growth factor binding protein, *SLC16A1 *Solute carrier family 16 member 1, *DRA *Down-regulated in adenoma, *PATL1 *Putative anion transporter isoform 1, *HMGCS1 *3-Hydroxy-3-methylglutaryl-CoA synthase 1, *HMGCL *3-Hydroxymethyl-3-methylglutaryl- CoA lyase, *BDH1 *3-Hydroxybutyrate dehydrogenase 1, *BDH2 *3-Hydroxybutyrate dehydrogenase 2

### Western blotting

Total protein was extracted from rumen epithelium tissue using RIPA Lysis Buffer (Nuoyang Biotechnology Co., Ltd., HangZhou, China), and the protein concentration was determined with Pierce™ Bicinchoninic Acid (BCA) Protein Assay Kit (Thermo Fisher, MA, USA). Each protein sample (100 mg) was combined with loading buffer and denatured at 100 °C for 5 min. Subsequently, protein samples (50 μg) and a dual color pre-stained broad molecular weight protein marker (Thermo Fisher, MA, USA) were separated using 10% SDS-PAGE. Separated proteins were transferred onto polyvinylidene fluoride (PVDF) membranes (Merck Millipore Corporation, USA). Following this, the membranes were blocked using StartingBlock™ (TBS) Blocking Buffer (Thermo Fisher, MA, USA) for 1 h at room temperature and incubated with a primary antibody for 14 h at 4 °C. The primary antibodies employed were rb-anti-cyclin D1 (Abcam, ab134175, 1:50,000 dilution), rb-anti-cyclin E1 (Abcam, ab33911, 1:2,000 dilution), rb-anti-CDK2 (Abcam, ab32147, 1:2,000 dilution), rb-anti-CDK4 (Abcam, ab199728, 1:2,000 dilution), rb-anti-CDK6 (Abcam, ab124821, 1:50,000 dilution), rb-anti-C-caspase 3 (Proteintech Group, 19677-1-AP, 1:500 dilution), rb-anti-C-caspase 7 (Proteintech Group, 27155-1-AP, 1:500 dilution), rb-anti-Bax (Servicebio, GB11690-100, 1:500 dilution), and mouse-anti-Cytc (Servicebio, GB12080-100, 1:2,000 dilution). After multiple washes with TBST, the membranes were incubated with goat anti-rabbit IgG HRP-conjugated secondary antibody (Fcmacs Biotechnology, Nanjing, China; 1:5,000) for 1 h at room temperature. Subsequently, the membranes were exposed to an HRP-conjugated mouse monoclonal GAPDH antibody (Santa Cruz, sc-32233, California, USA; 1:200) for normalization. Protein bands were visualized and photographed using an enhanced chemiluminescence reaction kit (Thermo Scientific) according to the manufacturer’s instructions and a Tanon 5500 Imager (Tanon, Shanghai, China). The density of the bands on the blot was analyzed using ImageJ software (National Institutes of Health, Bethesda, MD, USA).

### Terminal deoxynucleotidyl transferase dUTP nick-end labeling (TUNEL) staining

The TUNEL staining assay kit (ab206386, Cambridge, UK) was utilized in accordance with the manufacturer’s instructions for in situ cell death detection. Paraffin sections were deparaffinized, rehydrated, and rinsed with PBS prior to the addition of the TUNEL reaction solution and Converter-POD. Endogenous peroxidase activity was blocked using H_2_O_2_ in methanol, followed by permeabilization with 1 g/L Triton X-100 in 0.1% sodium citrate. Cells with brown granules in the nucleus were considered apoptotic. The authors conducting the study were blinded to the animal grouping. Slices were systematically photographed in a non-overlapping manner, moving from left to right and top to bottom. Each picture was assigned a number, and random fields were selected using a computer-generated random number method. The apoptotic rate was determined by counting the number of TUNEL-positive cells compared with all visible cells at 400× magnification. The mean value was calculated from a minimum of eight measurements, and the final values were analyzed.

### Transmission electron microscopy

The rumen epithelium tissue was fixed overnight with 2.5% glutaraldehyde (Solarbio, Beijing, China) in PBS at 4 °C. Following three 10-min washes with PBS, the tissues were cut into small pieces (approximately 1 mm^3^) and post-fixed with filtered 2% OsO_4_ in 30 mmol/L HEPES buffer (pH 7.4) containing 100 mmol/L NaCl and 2 mmol/L CaCl_2_ for 1 h at 4 °C with rotation. Subsequently, the samples were dehydrated and embedded, and 60-nm thin sections were mounted on copper grids. The ultrathin sections were observed under a transmission electron microscope (JEM-1400, JEOL Ltd., Tokyo, Japan).

### Statistical analysis

Data are expressed as mean ± standard deviation (SD), and statistical significance was set at *P* < 0.05. Data from the two groups were compared using Student's *t*-test to determine significant differences between means. Statistical analyses were performed using GraphPad Prism 8 software (version 18.0; SPSS Inc., Chicago, IL, USA).

## Results

### Animal performance and ruminal fermentation

**Fig. 2:**
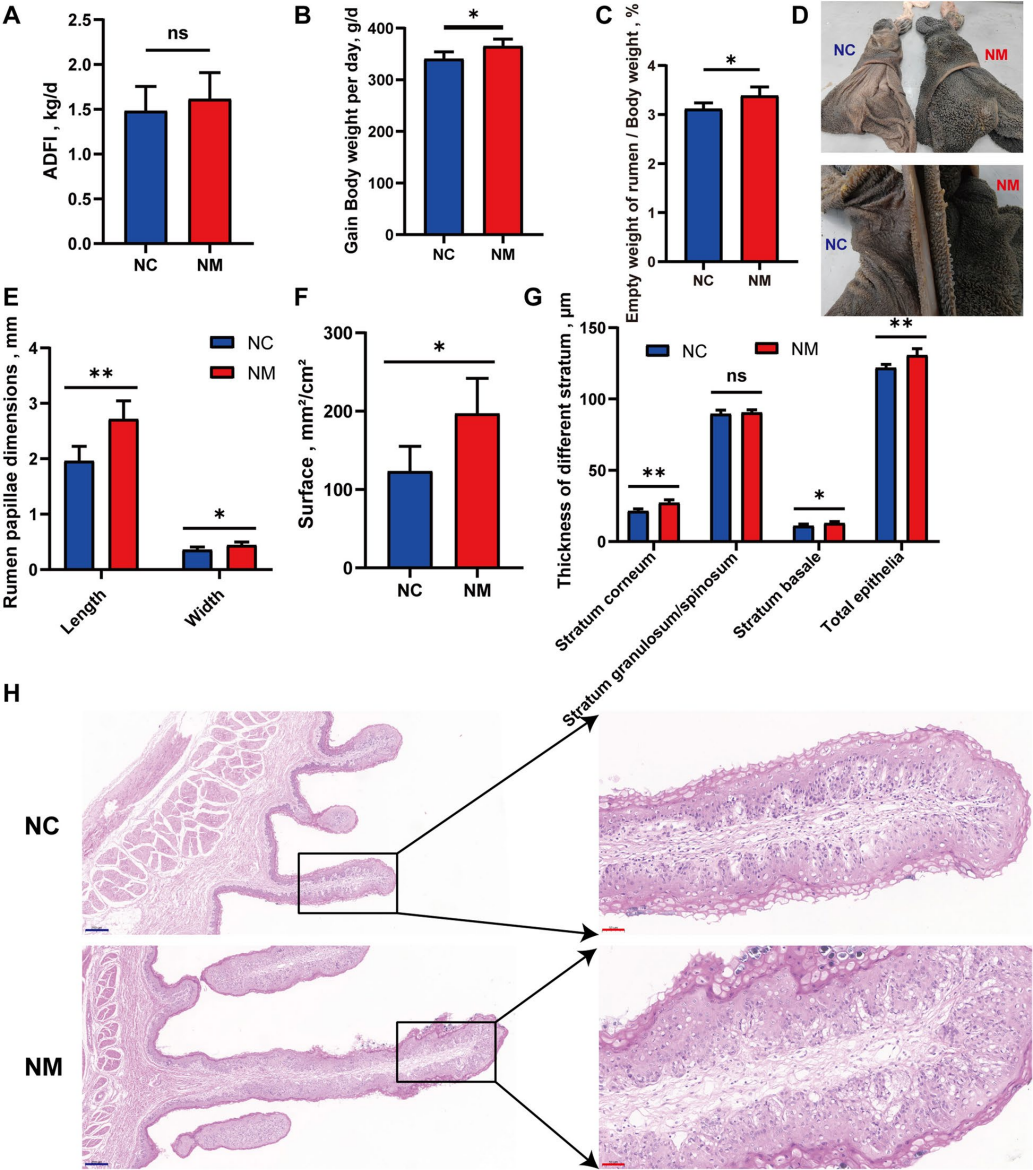
Effects of composite yeast culture on daily feed intake, weight gain, and rumen epithelial growth. **A **Average daily feed intake. **B **Average daily weight gain. **C **The percentage of empty rumen weight to body weight. **D **Organoleptic rumen images. **E **Rumen papillae length and width. **F **Rumen papillary surface area. **G **Rumen epithelial morphology. **H **Rumen epithelial photomicrographs (H&E; Blue scale bar = 50 µm, Red scale bar = 200 µm). Data are presented as mean ± SD. Different symbols above the bars indicate the level of significance: ^*^P < 0.05, ^**^P < 0.01. ADFI, average daily feed intake; NC, basal diet control group; NM, basal diet + composite yeast culture

All animals remained healthy throughout the feeding trials. Figure 2A shows no significant change in the average daily feed intake of lambs in the NM group (*P* = 0.075). Yeast culture supplementation significantly enhanced lamb growth performance: the NM group exhibited a 58.98 g/d increase in average daily weight gain (*P* < 0.05; Fig. 2B), corresponding to an 18.69% improvement in final body weight. Furthermore, the rumen empty weight-to-final body weight ratio increased by 0.27% (*P* < 0.05; Fig. 2C), indicating improved rumen development relative to overall growth. Collectively, these findings highlight the ability of the yeast culture to optimize both growth efficiency and rumen-to-body mass proportionality in lambs.

We examined the ruminal fermentation patterns of lambs. Lambs in the NM group exhibited a significant decrease in rumen pH by 0.36 compared with the NC group (*P* < 0.01), along with a notable increase of 8.35% in total volatile fatty acids (*P* < 0.01) (Table 4). Furthermore, the concentrations of acetate, propionate, butyrate, isobutyrate, valerate, and isovalerate showed significant increases (*P* < 0.05), whereas the acetate ratio significantly decreased by 3.88% (*P* < 0.01). In addition, the proportions of butyrate and valerate significantly increased by 2.37% (*P* < 0.01) and 0.53% (*P* < 0.05), respectively. There were no significant changes in the proportions of propionate, isobutyrate, and isovalerate.

**Table 4:** Effect of complex yeast culture on rumen pH and VFA concentration in sheep

Items	NC	NM	*P*-value
Ruminal pH	6.87 ± 0.17	6.51 ± 0.26	< 0.0001
Total VFA, μg/mL	3160.36 ± 35.80	3424.37 ± 47.49	< 0.0001
Acetate, μg/mL	1855.36 ± 11.53	1877.58 ± 13.69	0.024
Propionate, μg/mL	594.87 ± 19.81	646.35 ± 21.11	0.004
Butyrate, μg/mL	465.18 ± 15.49	585.41 ± 16.84	< 0.0001
Isobutyrate, μg/mL	77.72 ± 6.77	96.29 ± 14.52	0.032
Valerate, μg/mL	86.64 ± 8.78	112.23 ± 10.13	0.003
Isovalerate, μg/mL	80.59 ± 13.24	106.52 ± 12.29	0.012
Acetate ratio, %	58.71 ± 0.36	54.83 ± 0.33	< 0.0001
Propionate ratio, %	18.82 ± 0.49	18.87 ± 0.42	0.866
Butyrate ratio, %	14.72 ± 0.47	17.09 ± 0.38	< 0.0001
Isobutyrate ratio, %	2.46 ± 0.21	2.81 ± 0.39	0.117
Valerate ratio, %	2.74 ± 0.27	3.27 ± 0.26	0.013
Isovalerate ratio, %	2.55 ± 0.41	3.12 ± 0.39	0.054

*NC *Basal diet control group, *NM *Basal diet+composite yeast culture, *VFA* Volatile fatty acids. Data are presented as mean ± SD

### Rumen papillae density, dimensions, surface area, and rumen epithelial morphology

To gain deeper insights into the effects of feed supplementation with yeast cultures on the development of the rumen epithelium in lambs, we assessed histological changes in the rumen papillae. The NM group, supplemented with yeast culture, exhibited a significant increase in the length, width, and surface area of the rumen papillae of the lambs (*P* < 0.05) (Fig. 2D–F). Conversely, no significant difference in the density of the rumen papillae was observed between the NM and control groups. The thicknesses of the sheep rumen epithelial cuticle and basal layer were significantly higher in the NM group (*P* < 0.05) (Fig. 2G). There was no significant difference in the thickness of the epithelial granular and sphenoid layers of the rumen between lambs from either group. Histological observations were similar to the above results (Fig. 2H).

### DEPs and their kinds

**Fig. 3:**
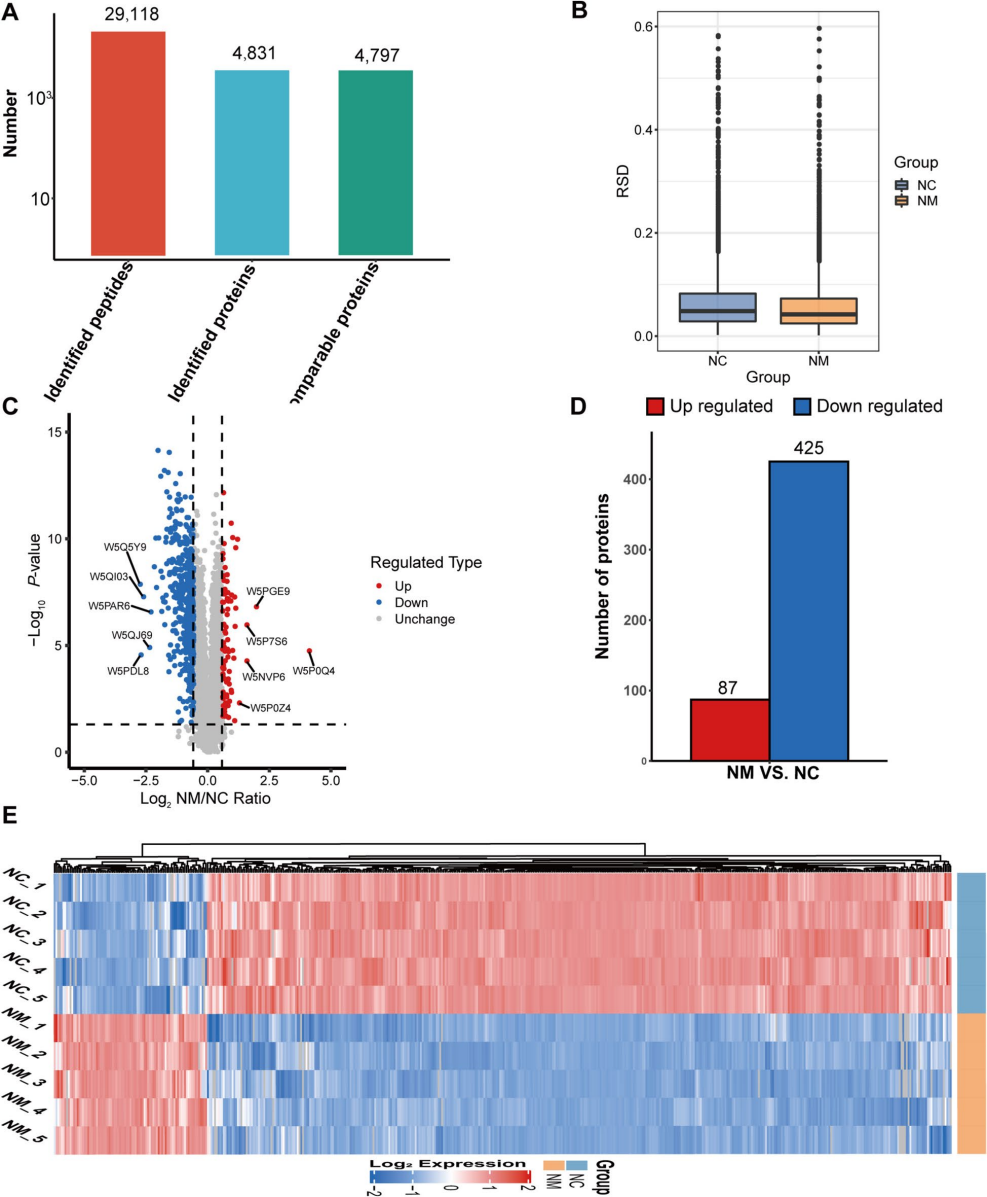
Effect of composite yeast culture on protein expression in sheep rumen epithelium. **A** Total number of identified proteins. **B** Relative standard deviation (RSD) plots. **C** Differential protein volcano plots. **D** Differential protein bar charts. **E** Differential protein heat maps. NC, basal diet control group; NM, basal diet + composite yeast culture group

A total of 4,831 proteins were identified using label-free LC–MS/MS, with a 1% false discovery rate. Figure 3A shows the overall numbers of peptides and proteins identified after filtering the search library data. Quantitative results of duplicate biological samples were examined for statistical consistency. The results of the relative standard deviation (RSD) obtained by calculating the relative quantitation values based on the relative quantitation values of each group of duplicate samples showed that the RSD value of each group of duplicate samples was < 0.1 (Fig. 3B). This consistency aligns with stringent quality control standards for analytical repeatability. For all identified proteins, the volcano map provided a clear visual representation of the relationship between NM and NC (Fig. 3C). Following analysis and identification, 512 DEPs were identified in the rumen epithelium of the two groups (Additional files 1 and 2). Figure 3D shows the 87 upregulated and 425 downregulated proteins. The DEPs in all comparison groups were plotted as an expression heat map (Fig. 3E). The results showed the relative expression levels of multiple DEPs in different samples and their clustering relationships in terms of relative expression. Briefly, the most upregulated proteins in lambs in the NM group included haptoglobin, alpha-1-acid glycoprotein, transmembrane protein 168 (TMEM168), sodium/hydrogen exchanger (SLC9A1), and apolipoprotein B (APOB). The most downregulated proteins in the NM group lambs included NADH dehydrogenase 1 beta subcomplex subunit 10 (NDUFB10), 5′-deoxynucleotidase (HDDC2), tropomyosin 1 (TPM1), serine protease 42 (PRSS42P), and prefoldin subunit 1 (PFDN1).

### The enrichment analysis of DEPs according to GO

**Fig. 4:**
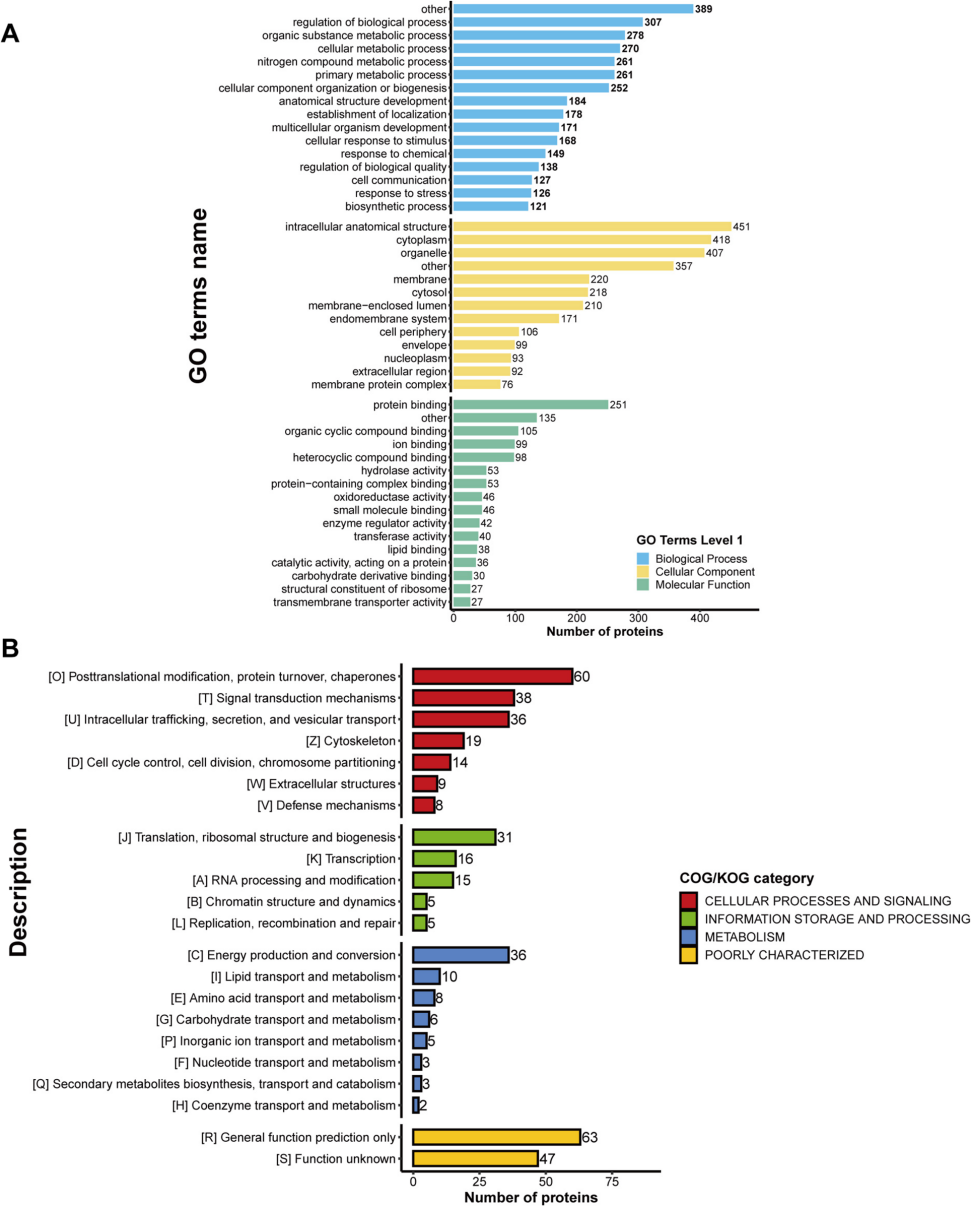
Functional classification of differential proteins in sheep rumen epithelium in the NC and NM groups. **A **GO secondary classification bar graph. **B **KOG functional classification bar graph. NC, basal diet control group; NM, basal diet + composite yeast culture group

The DEPs were significantly enriched in 45 terms, of which 16 were annotated with molecular function (MF), 13 with cellular components (CC), and 16 with biological processes (BP) (Fig. 4A, Additional file 3). The top five biological progresses were mitochondrial respiratory chain complex assembly (GO:0006119), mitochondrial translational elongation (GO:0033108), mitochondrial translational termination (GO:0070125), and translation termination transmembrane transport (GO:0070126). The molecular functions had the following five most significant GO terms: NADH dehydrogenase (ubiquinone) activity (GO:0008137), NADH dehydrogenase (quinone) activity (GO:0050136), NADH dehydrogenase activity (GO:0003954), glutathione transferase activity (GO:0004364), and glutathione binding (GO:0043295). As for cell components, mitochondrial protein-containing complex (GO:0098798), mitochondrial inner membrane (GO:0005743), organelle inner membrane (GO:0019866), inner mitochondrial membrane protein complex (GO:0098800), and respiratory chain complex (GO:0098803) were the top five GO terms significantly enriched (*P* < 0.05) (Fig. 4B, Additional file 4). Overall, we found that feeding yeast culture had a significant effect on mitochondrial function in rumen epithelial cells.

### KEGG pathway enrichment analysis of DEPs

**Fig. 5:**
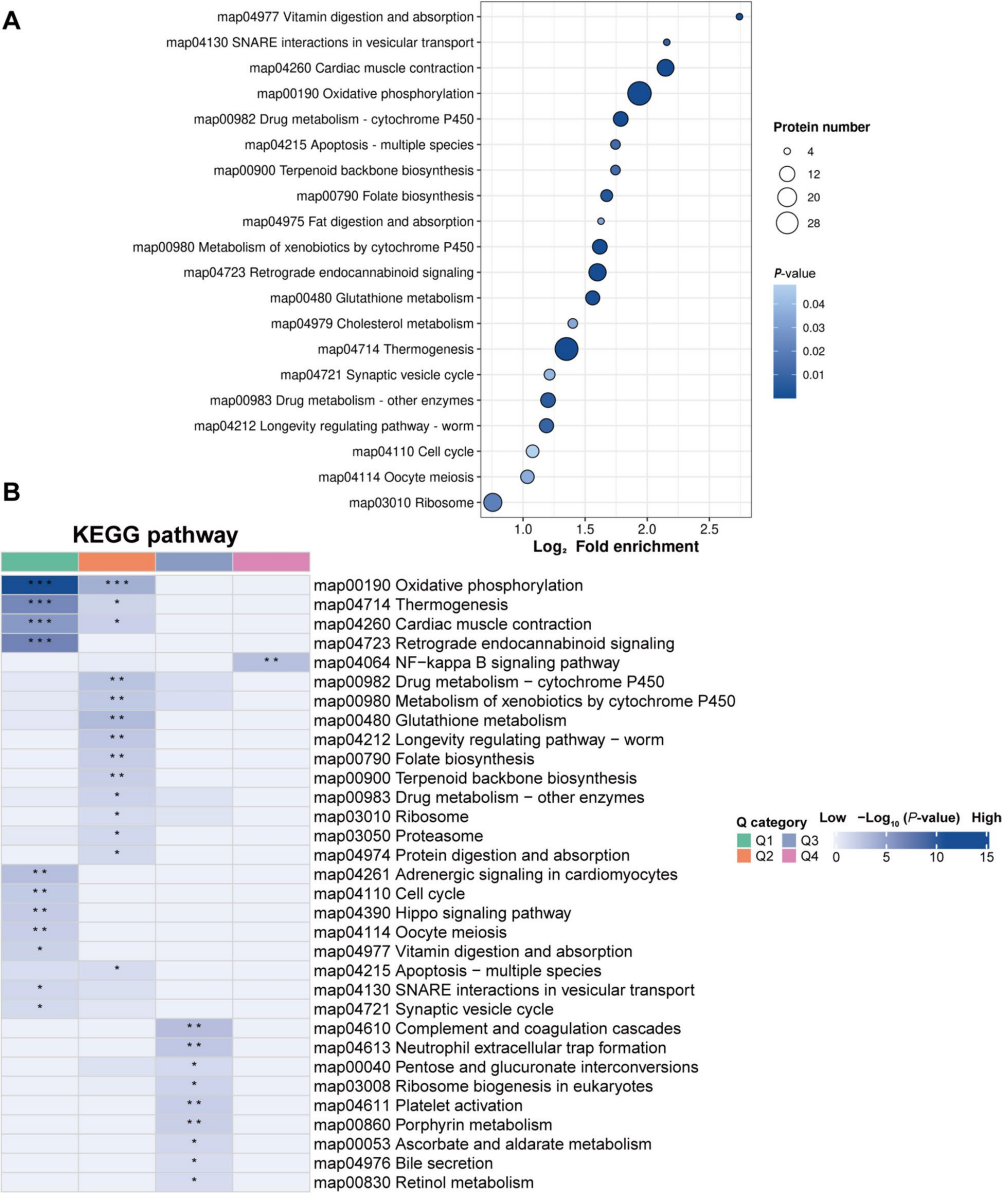
Functional enrichment analysis of DEPs in sheep rumen epithelium in the NC and NM groups. **A** KEGG pathway enrichment bubble plot (the color of the dots represents the *P*-value of enrichment significance; the bluer the color, the stronger the enrichment significance; the size of the dots represents the number of differential proteins in the KEGG pathway; the larger the dots, the more differential proteins in the pathway). **B **KEGG pathway clustering analysis. Blue indicates high enrichment significance; blue and white indicate low enrichment significance. ^*^P < 0.05, ^**^P < 0.01, ^***^P < 0.0001. NC, basal diet control group; NM, basal diet + composite yeast culture group; DEPs, differentially expressed proteins

We next investigated the effects of DEPs on pathways related to yeast culture by analyzing the KEGG pathway enrichment. Twenty pathways were enriched in DEPs of the NM and NC groups (Fig. 5A, Additional file 5). The significantly enriched KEGG terms were Oxidative phosphorylation (map00190), Thermogenesis (map04714), Cardiac muscle contraction (map04260), Retrograde endocannabinoid signaling (map04723), Glutathione metabolism (map00480), Drug metabolism—cytochrome P450 (map00982), Metabolism of xenobiotics by cytochrome P450 (map00980), SNARE interactions in vesicular transport (map04130), Apoptosis (map04215), Folate biosynthesis (map00790), Longevity regulating pathway—worm (map04212), Vitamin digestion and absorption (map04977), Drug metabolism—other enzymes (map00983), Synaptic vesicle cycle (map04721), Proteasome (map03050), Oocyte meiosis (map04114), Terpenoid backbone biosynthesis (map00900), Collecting duct acid secretion (map04966), Cell cycle (map04110), and Ribosome (map03010).

To compare the functional similarities and differences between proteins with different differential ploidy, we divided the proteins into four fractions based on differential expression ploidy, Q1–Q4 (Q1: < 0.5; Q2: 0.5–0.667; Q3: 1.5–2.0; Q4: > 2.0) (Additional file 6), and performed KEGG enrichment for each fraction separately, followed by functional clustering analysis. Cluster analysis showed that the pathways with a high enrichment ratio (Fig. 5B, Additional file 7) included oxidative phosphorylation, retrograde endocannabinoid signaling, and Thermogenesis in Q1; Oxidative phosphorylation and glutathione metabolism in Q2; Complement and coagulation cascades and neutrophil extracellular trap formation in Q3; the NF-kappa B signaling pathway in Q4. However, the KEGG pathways of interest, such as cell cycle (Additional file 8) and apoptosis (Additional file 9), were clustered into Q1 and Q2, respectively. These data showed that proteins (YWHAE, YWHAB, YWHAH, YWHAG, and SFN) that negatively regulate the cell cycle were downregulated in the NM group. Furthermore, the downregulation of apoptosis-promoting proteins (CASP7, BAK1, CYCS, DIABLO, and BAX) was observed in the NM group. The results of this study revealed accelerated proliferation of rumen epithelial cells in chronically fed lambs and molecular evidence of attenuated apoptosis.

### PPI analysis of DEPs

**Fig. 6:**
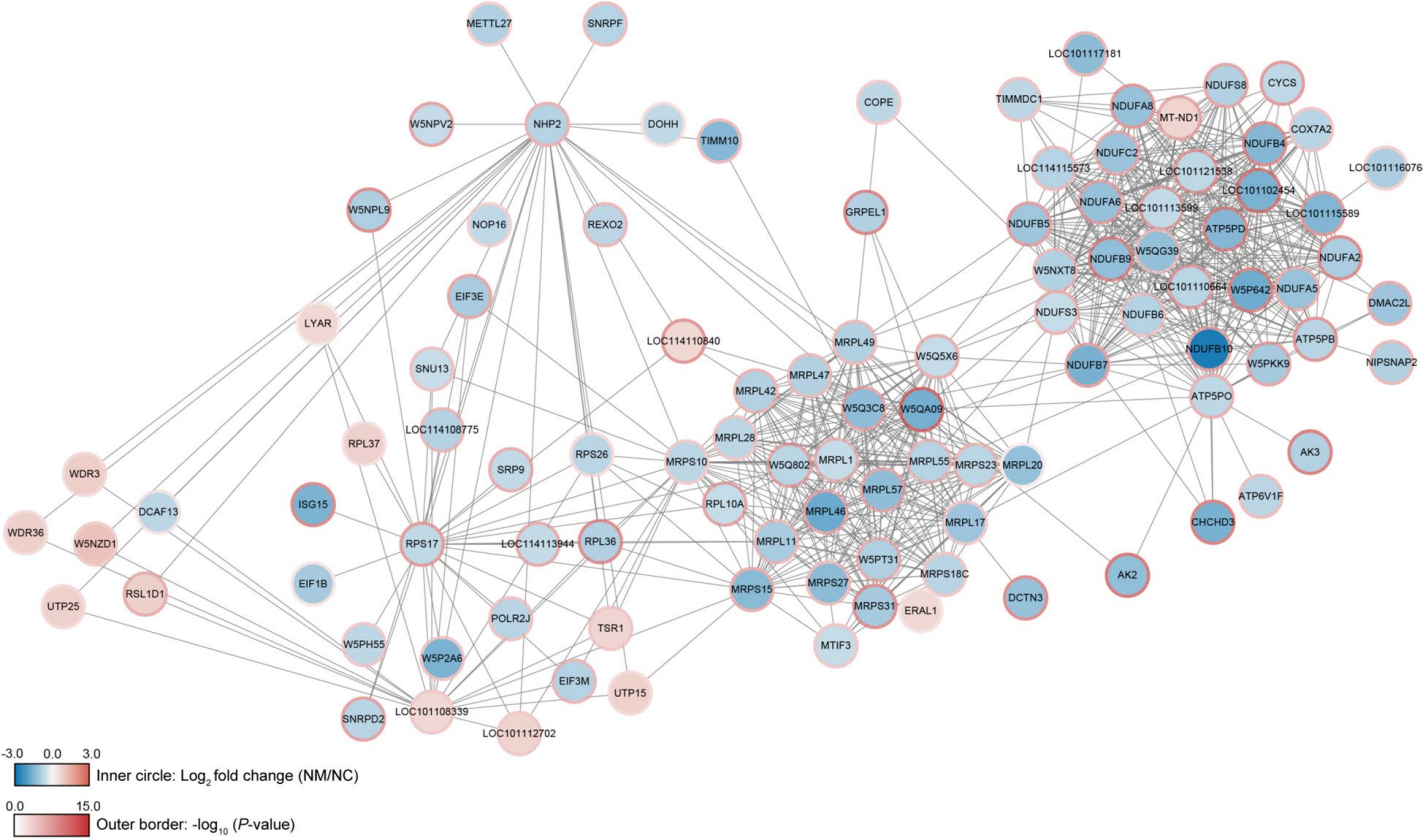
Interaction network analysis of DEPs in sheep rumen epithelium in the NC and NM groups. Circles indicate DEPs, different colors represent differential protein expressions (blue represents downregulated proteins, red represents upregulated proteins), the darker the color, the greater the difference, and the size of the circle indicates the number of proteins with which it interacts. NC, basal diet control group; NM, basal diet + composite yeast culture group; DEPs, differentially expressed proteins

We analyzed the network of STRING database-validated protein–protein interactions of DEPs. Fourteen upregulated and 89 downregulated DEPs met the minimum interaction score requirements (Fig. 6, Additional file 10). The proteins with the highest PPI were MRPS10 (degree: 33), ATP5PO (degree: 31), W5NXT8 (degree: 31), and NDUFB7 (degree: 30) (Additional file 10). There were four distinct subnetworks based on biological functions. The upregulated proteins in the NM group were involved in ribosome biogenesis in eukaryotes, whereas the downregulated proteins were associated with oxidative phosphorylation and mitochondrial protein-containing complexes (Fig. 6). Notably, several DEPs in the networks, including MTIF3, W5PT31, MRPS27, MRPS23, MRPS18C, MRPL57, MRPL55, MRPL47, W5Q3C8, MRPL42, W5Q802, W5Q5X6, MRPL20, W5QA09, MRPL11, MRPL1, and TIMM10, were associated with mitochondria.

### Cell cycle analysis of rumen epithelial tissues

**Fig. 7:**
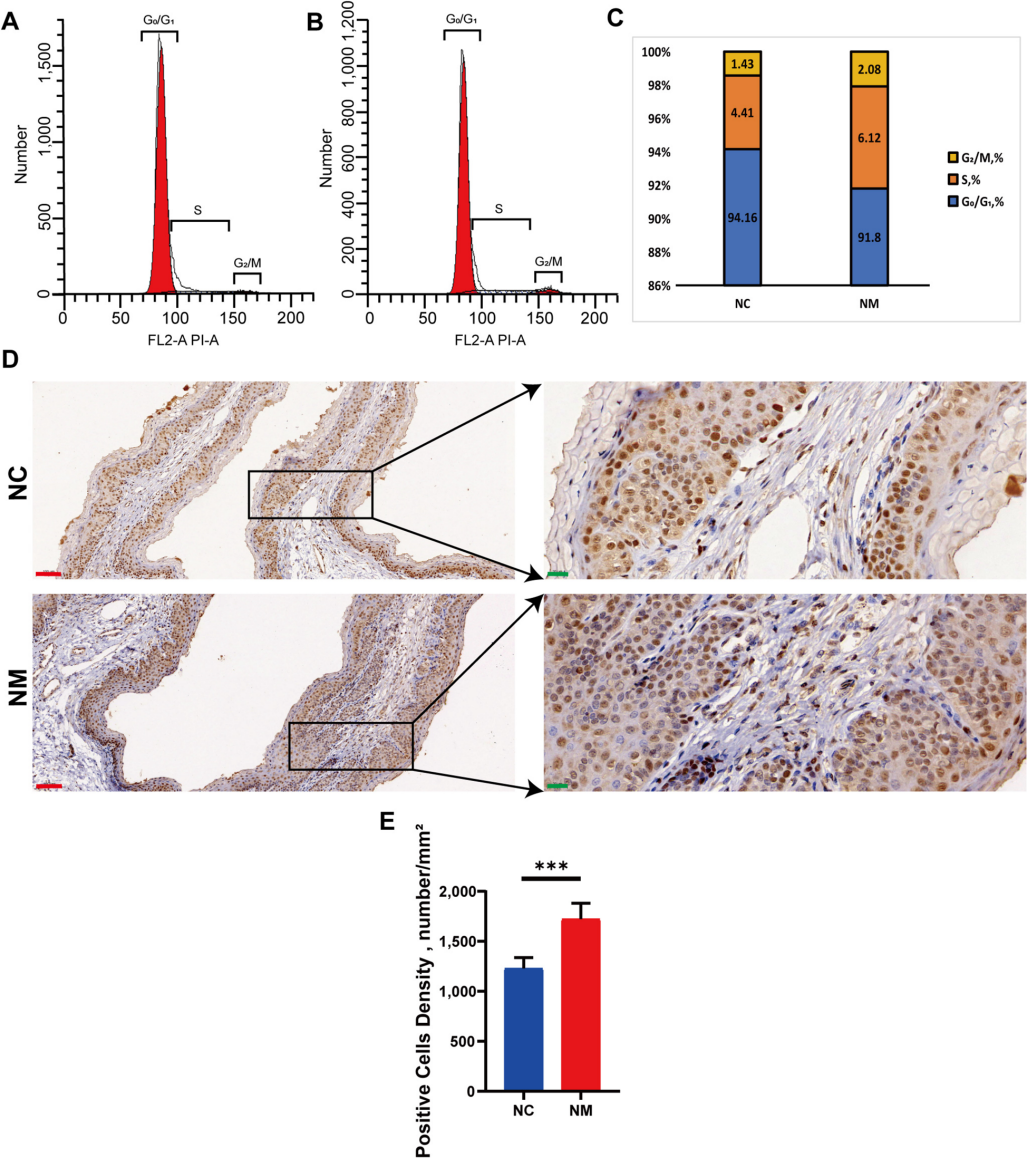
Effect of complex yeast culture on cell cycle and PCNA in sheep rumen epithelial cells. **A **Cell cycle in the NC group. **B **Cell cycle progression in the NM group. **C **Percentage stacked plots of the cell cycle in the NC and NM groups. **D **PCNA plot of the NC and NM groups (Red scale bar = 20 µm, Green scale bar = 100 µm). **E **Histogram of PCNA-positive cell numbers. Data are presented as mean ± SD. ^***^P < 0.001. G_0_/G_1_: ratio of cells in the quiescent and growth phases; S: ratio of cells in the DNA synthesis phase; G_2_/M: ratio of cells in the division phase (the percentage was based on 10,000 cells measured). NC, basal diet control group; NM, basal diet + composite yeast culture group; PCNA, proliferating cell nuclear antigen

To determine the growth of rumen epithelial cells, we used flow cytometry to identify cell cycle stages in the NC and NM groups. Figures 7A–C show that the proportion of rumen epithelial cells in the G_0_/G_1_ phase was significantly lower in the NM than NC groups (*P* < 0.05), whereas the proportion of cells in the S phase was significantly higher (*P* < 0.05). The proportion of cells in the G_2_/M phase increased by 0.65%; however, this difference was not significant. These results indicate that yeast culture accelerates the G_1_ phase of the ruminal epithelial cell cycle and encourages cell growth. The proliferative state of cells can be objectively evaluated using proliferating cell nuclear antigens (PCNA). PCNA protein was detected in the rumen tissues of the NM and NC groups using specific staining. The NM group contained more PCNA-positive cells than the NC group (Fig. 7D). Semi-quantitative analysis showed that PCNA was present in the nucleus, with a positivity rate of 1,229 numbers/mm^2^ in NM tissues and 1,722 numbers/mm^2^ in NC tissues, which showed significant differences (Fig. 7E). Feeding yeast culture decreased the proportion of cells in the G_0_/G_1_ phase and increased the proportion of cells in the S and G_2_/M phases.

These results indicate that feeding yeast culture shortens the G_0_/G_1_ phase of rumen epithelial cells and accelerates the cell cycle, promoting the proliferation of ruminal epithelial cells.

### Expression of genes and proteins involved in cell cycle regulation in rumen epithelium

**Fig. 8:**
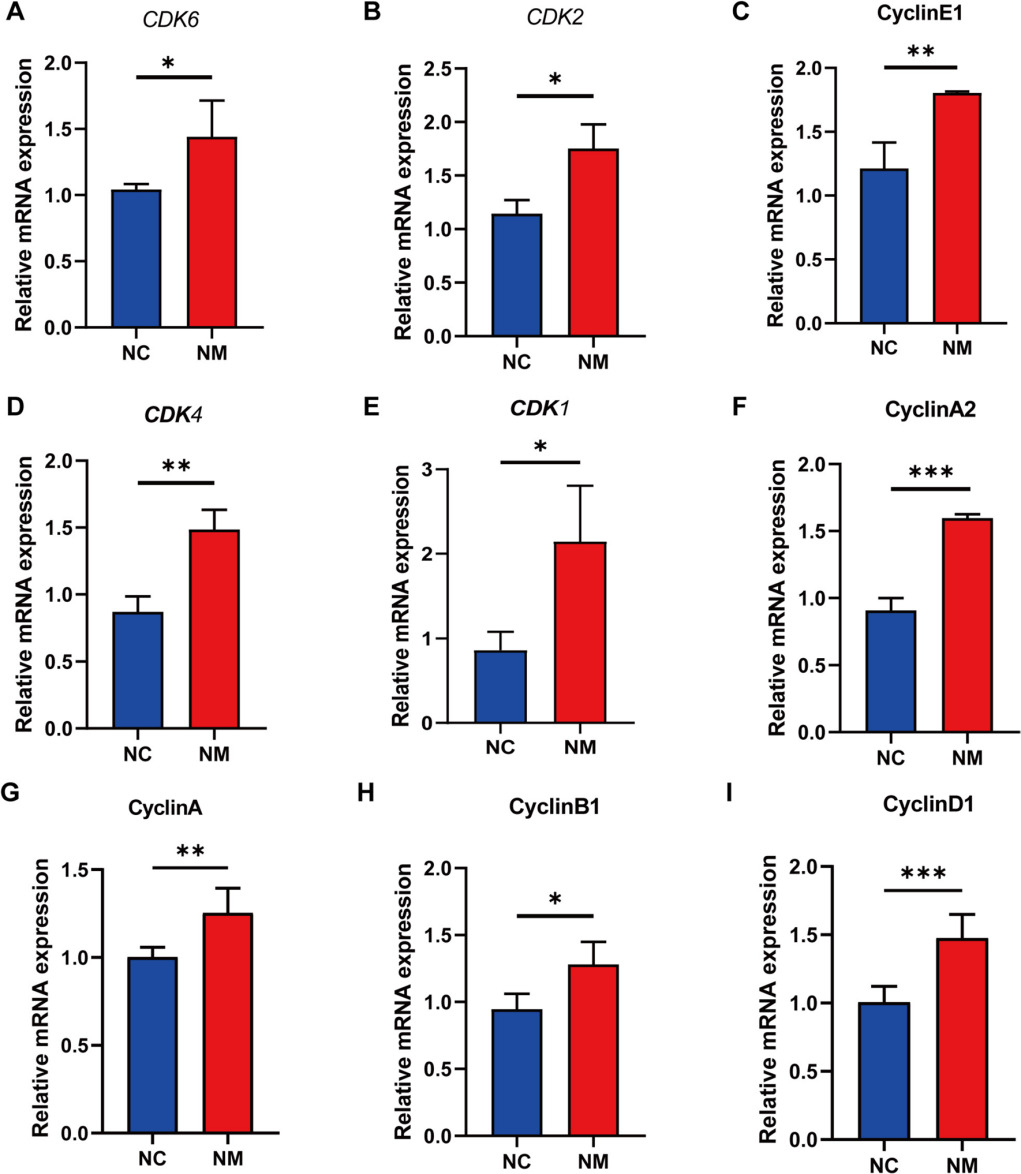
Complex yeast culture effects on cyclins and cyclin-dependent protein kinase gene expression in ovine rumen epithelial cells. **A **CDK6. **B **CDK2. **C **Cyclin E1. **D **CDK4. **E **CDK1. **F **Cyclin A2. **G **Cyclin A. **H ** Cyclin B1. **I **Cyclin D1. Data are expressed as mean ± SD. Different symbols above the bars indicate the level of significance: ^*^P < 0.05, ^**^P < 0.01, and ^***^P < 0.001. NC, basal diet control group; NM, basal diet + composite yeast culture group; CDK, cyclin-dependent kinase

**Fig. 9:**
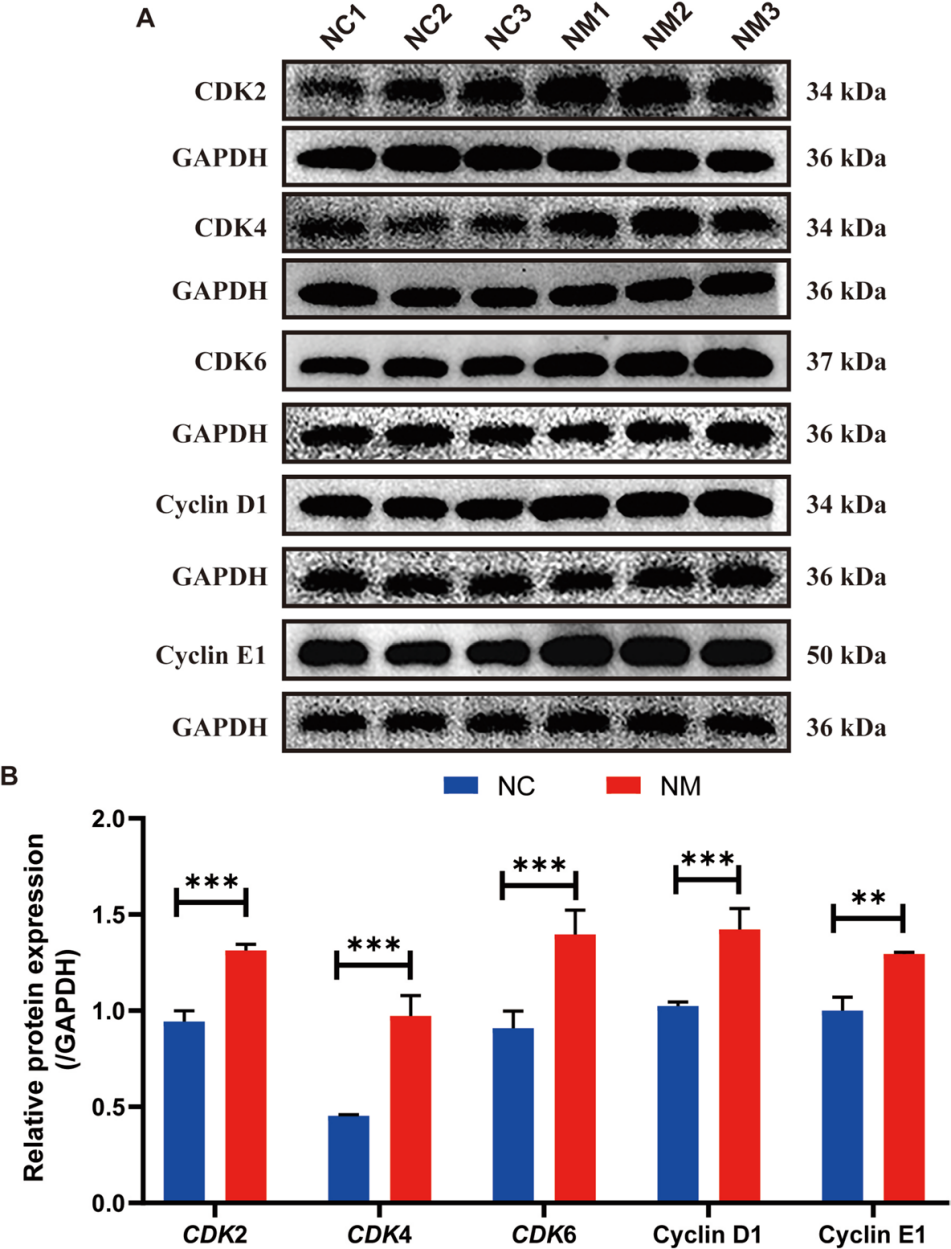
Composite yeast culture effects on cyclins and cyclin-dependent protein kinase gene expression in ovine rumen epithelial tissue. **A **Protein extracts from rumen epithelial samples were prepared and immunoblotted using specific antibodies. **B **The intensities of CDK2, CDK4, CDK6, cyclin D1, and cyclin E1 were normalized to the corresponding GAPDH levels. Data are expressed as mean ± SD. Different symbols above the bars indicate the level of significance: *P* < 0.01, ^***^P < 0.001. NC, basal diet control group; NM, basal diet + composite yeast culture group; CDK, cyclin-dependent kinase

In the eukaryotic cell cycle, cyclins D1, D2, D3, E1, and E2 are positive regulators that control the transition from the G_0_/G_1_ phase to the S phase, whereas cyclin-dependent kinase inhibitors 1A, 2A, 1B, and 2B control the negative regulators. Figure 8 shows the expression levels of genes encoding cell cycle-regulating proteins (cyclin D1, cyclin A2, cyclin E1, cyclin A, cyclin B1, *CDK1*, *CDK2*, *CDK4*, and *CDK6*). Cyclin and *CDK* expressions were significantly increased in the NM group. To determine how yeast culture affect rumen epithelial cells, we examined cyclin D1, CDK2, CDK4, CDK6, and cyclin E1 expression using western blotting. Figures 9A and B show that cyclin D1, CDK2, CDK4, CDK6, and cyclin E1 levels were higher in the NM group. These results indicate that yeast culture affects rumen epithelial cells by regulating cell cycle-related proteins.

### TUNEL and ultrastructure of the rumen epithelium

**Fig. 10:**
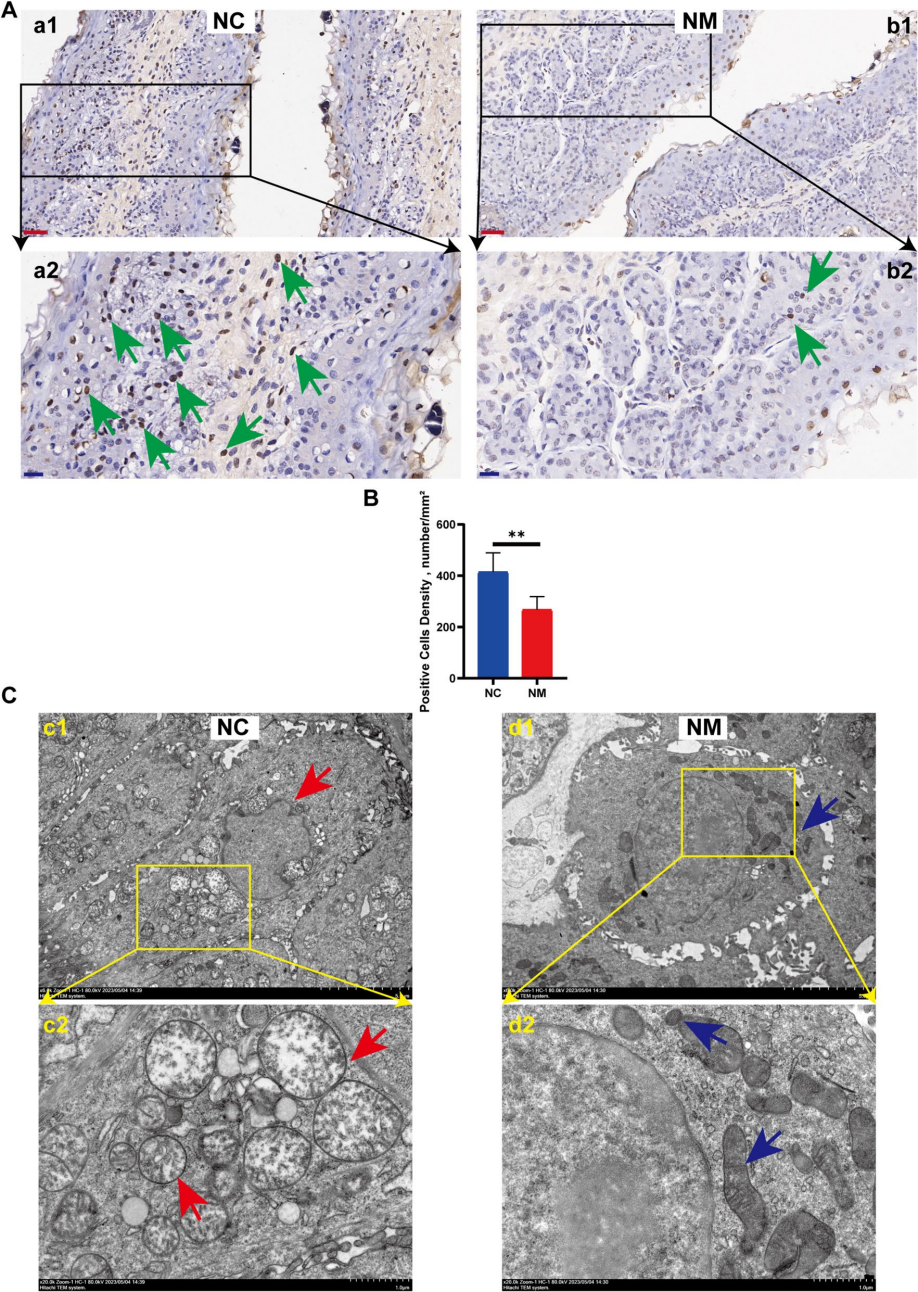
Effect of complex yeast culture on apoptosis of sheep rumen epithelial cells. **A **TUNEL plots of NC and NM groups; Red scale bar = 60 µm; Blue scale bar = 20 µm (green arrows indicate positive cells). **B **Histogram of TUNEL-positive cell numbers. **C **Ultrastructural map of rumen epithelial cells, C1 and D1 scale bar = 5.0 µm, C2 and D2 scale bar = 1.0 µm (red arrows indicate wrinkled and irregular nuclei, convex and uneven nuclear membrane surfaces, swollen and enlarged mitochondria, cristae fusion, disappearance, or breakage; blue arrows indicate typical nuclear and mitochondrial structures). Data are expressed as mean ± SD. ^**^P < 0.01. NC, basal diet control group; NM, basal diet + composite yeast culture group

We conducted TUNEL staining of rumen epithelium in lambs from the NM and NC groups. The NC group exhibited a higher apoptotic percentage than the NM group (Fig. 10A and B), and the apoptotic percentage significantly decreased following the administration of yeast culture. To further examine ultrastructural alterations in rumen epithelial tissue, transmission electron microscopy (TEM) was performed on lambs from the NM and NC groups. The findings indicated that certain cells within the rumen epithelium of the NC group displayed shrunken and irregularly shaped nuclei, along with uneven nuclear membranes. In addition, the mitochondria were significantly enlarged and the mitochondrial cristae were fused, disappeared, or broken. In contrast, ruminal epithelial cells in the NM group exhibited typical nuclear and mitochondrial structures (Fig. 10C). These findings indicate that yeast culture can mitigate apoptosis in rumen epithelial cells, which facilitates the proliferation of these cells.

### Expression of genes and proteins involved in apoptosis regulation in rumen epithelium

**Fig. 11:**
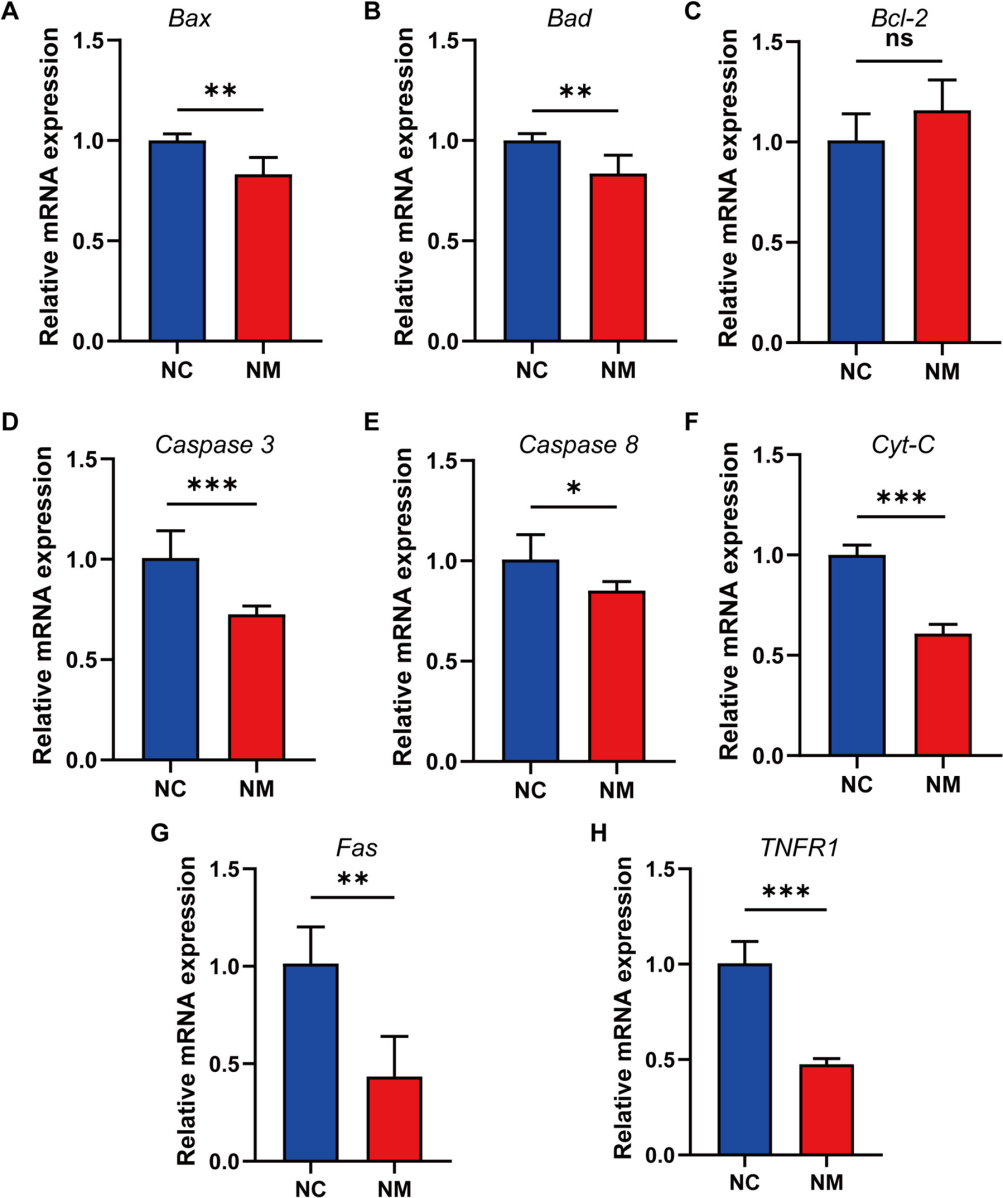
Composite yeast culture effects on the expression of genes that regulate apoptosis in ovine rumen epithelial tissues. **A **Bcl-2-associated X protein (*Bax*). **B **Bcl-2 asociated death promoter (*Bad*). **C **B-cell lymphoma-2 (*Bcl-2*). **D **Cysteinyl aspartate-specific proteinase 3 (*caspase 3*). **E **Cysteinyl aspartate-specific proteinase 8 (*caspase 8*). **F **Cytochrome Complex (*Cyt-c*). **G **Factor-related Apoptosis (*Fas*). **H **Tumor Necrosis Factor Receptor 1 (*TNFR1*). Data are expressed as mean ± SD. Different symbols above the bars indicate the level of significance: ^*^P < 0.05, ^**^P < 0.01, ^***^P < 0.001. NC, basal diet control group; NM, basal diet + composite yeast culture group

**Fig. 12:**
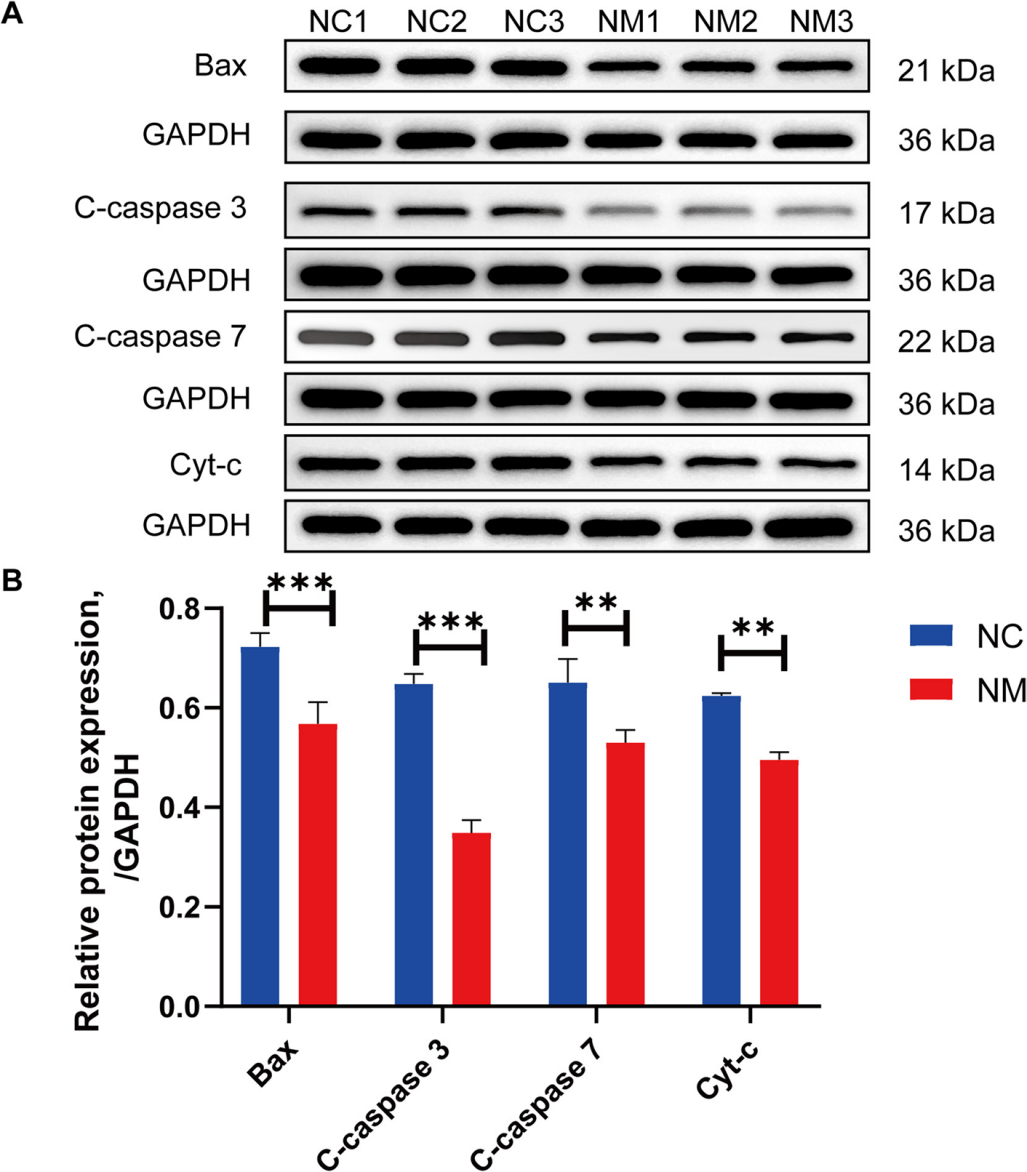
Effect of composite yeast culture on the protein expression of genes involved in apoptosis regulation in sheep rumen epithelial tissue. **A **Protein extracts from rumen epithelial samples were prepared and immunoblotted with specific antibodies. **B** The intensities of Bax, cleaved caspase (C-caspase) 3, C-caspase 7, and Cyt-c were normalized to the corresponding GAPDH levels. Data are expressed as mean ± SD. Different symbols above the bars indicate the level of significance: ^**^P < 0.01, ^***^P < 0.001. NC, basal diet control group; NM, basal diet + composite yeast culture group

To investigate the effect of yeast culture on apoptosis-related gene expressions in the rumen epithelium of lambs, we detected key genes involved in the apoptotic pathway using fluorescence quantitative (q)PCR and western blot. Cytochrome C (*Cyt-c*), Bcl-2 associated X protein (*Bax*), Bcl-2 associated death promoter (*Bad*), *caspase 3*, *caspase 8*, *Fas*, and *TNFR1* expressions were significantly reduced (*P* < 0.05) in the NM group compared with the NC group (Fig. 11). However, the difference in *Bcl-2* expression between the two groups was not significant (Fig. 11C). Furthermore, Cyto-c, Bax, cleaved caspase 3 (C-caspase 3), and cleaved caspase 7 (C-caspase 7) protein expression was significantly reduced (*P* < 0.05) in the NM group compared with the NC group (Fig. 12). These results indicate that yeast culture influence the proliferation of rumen epithelial cells by modulating apoptosis-related proteins.

### Expression of genes associated with the insulin-like growth factor 1 (IGF-1) signaling pathway and metabolism of volatile fatty acids in the rumen epithelium

**Fig. 13:**
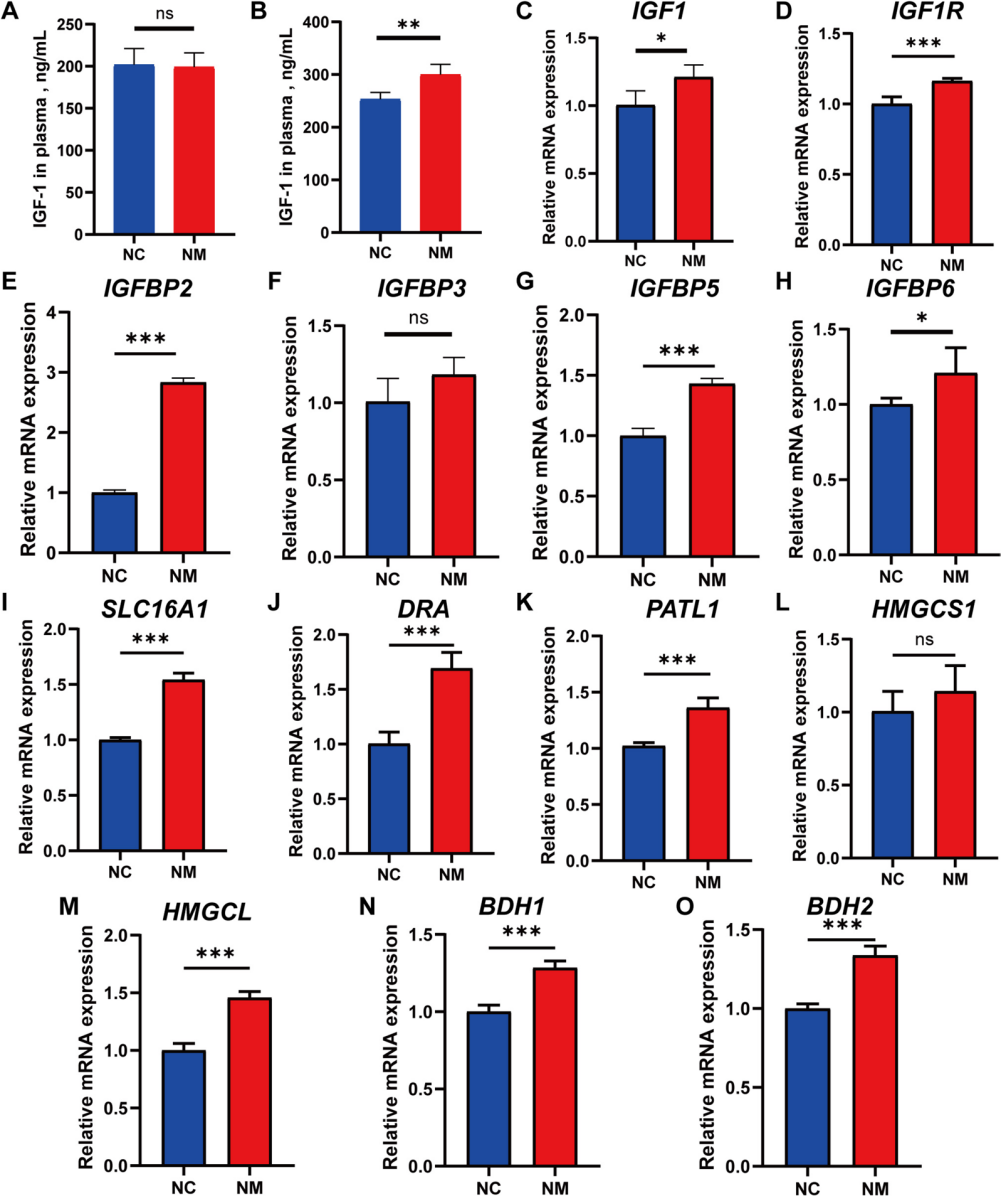
Complex yeast culture effects on serum IGF-1 and expression of genes related to VFA metabolism. **A **Serum levels of insulin-like growth factor 1 (IGF-1) in sheep at the start of the trial. **B **Serum levels of IGF-1 in sheep at the end of the experiment. **C ***IGF1* gene expression levels in the rumen of sheep in different groups. **D ***IGF1R* gene expression levels in the rumen of sheep in different groups. **E ***IGFBP2* gene expression levels in the rumen of sheep in different groups. **F ***IGFBP3* gene expression levels in the rumen of sheep in different groups. **G ***IGFBP5* gene expression levels in the rumen of sheep in different groups. **H ***IGFBP6* gene expression levels in the rumen of sheep in different groups. **I ***SLC16A1* gene expression levels in the rumen of sheep in different groups. **J ***DRA* gene expression levels in the rumen of sheep in different groups. **K ***PATL1* gene expression levels in the rumen of sheep in different groups. **L ***HMGCS1* gene expression levels in the rumen of sheep in different groups. **M ***HMGCL* gene expression levels in the rumen of sheep in different groups. **N ***BDH1* gene expression levels in the rumen of sheep in different groups. **O ***BDH2* gene expression levels in the rumen of sheep in different groups. Data are expressed as mean ± SD. Different symbols above the bars indicate the level of significance: ^*^P < 0.05, ^**^P < 0.01, ^***^P < 0.001. NC, basal diet control group; NM, basal diet + composite yeast culture group

Considering the pivotal role of IGF-1 signaling pathway in regulating rumen epithelial growth, we investigated the concentration of IGF-1 in the plasma of lambs. The initial observations indicated that the discrepancy between the NM and NC groups was not significant (Fig. 13A). After one month of consistent yeast culture feeding, the NM group exhibited a significantly higher plasma IGF-1 concentration than the NC group. (Fig. 13B).

Furthermore, the mRNA expression levels of *IGF1*, *IGF1R*, *IGFBP2*, *IGFBP3*, *IGFBP5*, and *IGFBP6* were evaluated using fluorescence qPCR. *IGF1* (Fig. 13C), *IGF1R* (Fig. 13D), *IGFBP2* (Fig. 13E), *IGFBP5* (Fig. 13G), and *IGFBP6* (Fig. 13H) mRNA expressions were higher in the NM than in the NC group. In addition, the difference in *IGFBP3* expression between the NM and NC groups was not significant (Fig. 13F). Absorption and metabolism of VFA are of great importance for the development and function of rumen epithelial cells. In this study, the genes related to VFA absorption and metabolism were identified using real-time fluorescence qPCR. The mRNA expression levels of *SLC16A1* (Fig. 13I), *DRA* (Fig. 13J), *PATL1* (Fig. 13K), *HMGCS1* (Fig. 13L), *HMGCL* (Fig. 13M), *BDH1* (Fig. 13N), and *BDH2* (Fig. 13O), which are involved in VFA absorption and metabolism, were increased in the rumen epithelial cells of lambs in the NM group.

## Discussion

The addition of yeast culture to diets has been shown to effectively increase daily weight gain and feed conversion ratio in ruminants (cattle and sheep), as well as promote rumen development and improve rumen barrier function [3, 44–49]. These findings are consistent with our results. In this study, the addition of the yeast culture increased the daily weight gain of lambs, which may be because yeast culture polysaccharides, such as mannans and other active ingredients, promote rumen fermentation and intestinal microbial metabolism in ruminants, which improves their feed utilization and growth performance. The pH of the rumen and concentration of VFA are major indicators of rumen function and alterations in its internal environment. The addition of yeast culture to the calf diet has beneficial effects on the growth of animals and rumen and intestinal tract [50].

This is primarily achieved through the enhancement of rumen fermentation, facilitated by increased butyric acid production and promotion of rumen papillary growth and development [3]. Notably, our findings revealed a similar increase in butyrate and total VFA concentrations in the rumen fluid of lambs following the addition of yeast culture. This indicates that yeast culture enhances butyrate production by modulating rumen fermentation patterns, thereby facilitating the repair of rumen epithelial damage. The rumen papillae are small projections of the rumen mucosal epithelium that increase the rumen surface area and facilitate the absorption of VFA. The most significant factor in rumen development is the length of the rumen papillae, followed by the width of the papillae and the thickness of the rumen wall [51]. The present study demonstrated that including a yeast culture in lamb diets had a marked effect on the development of rumen epithelium, resulting in a notable increase in rumen papilla length. A well-developed rumen papilla is essential for effective nutrient absorption and utilization, including nutrient uptake, metabolism, rumen pH regulation, and immune and barrier functions required to maintain a healthy rumen in ruminants [52]. The observed pH reduction despite improved rumen epithelial development indicates a potential disequilibrium between VFA production and absorption kinetics. Yeast supplementation likely stimulated microbial fermentation, increasing VFA output beyond the immediate absorptive capacity of the epithelium. This aligns with findings that rumen pH stability depends on synchronized adaptation of both microbial activity and epithelial transport systems [49, 53]. The promotion of rumen development by yeast culture may be associated with its function in regulating the rumen microbiota to elevate VFA concentrations, as well as the role of yeast cell wall polysaccharides in adsorbing LPS and other toxins in the rumen. This prevents LPS and other toxins from entering the bloodstream through the ruminal epithelium, thereby preventing inflammation. However, the precise mechanism through which yeast culture promotes rumen development remains to be determined.

The rumen epithelium plays a pivotal role in ruminant metabolic processes and exhibits remarkable adaptive and proliferative capabilities. To date, data regarding the biological alterations in the rumen epithelium elicited by administration of yeast culture remain limited. A total of 512 DEPs were identified in the rumen epithelium of the NM and NC groups. The DEPs were associated with several biological processes, including organic substances and cellular metabolic processes, nitrogen compound and organic cyclic compound binding, ion binding, heterocyclic compound binding, and hydrolase activity. KEGG analysis of the DEPs revealed that 13 of the 32 significantly enriched metabolic-associated pathways, indicating that the functional differences between the rumen epithelia of the NM and NC groups were primarily concentrated in the metabolism of rumen epithelial cells. However, the most intriguing of these significantly enriched pathways were those associated with cell cycle and apoptosis, as they directly influence the morphological alterations of the rumen epithelium.

Enlarged rumen papillae may be associated with accelerated cell cycle progression [54, 55]. The cell cycle drives cells to divide, yielding two new daughter cells. During this process, proliferating cells sequentially transition through the G_1_, S, G_2_, and M phases for DNA synthesis, cell division, and mitosis [56]. However, in some cases, cells exit the cell cycle and enter the G0 phase, which is a quiescent state. These dormant cells possess minimal cell cycle machinery and maintain specific cellular functions rather than undergoing cell proliferation [57].

In this study, we employed flow cytometry to analyze DNA content, which was used to assess the cell cycle state of rumen epithelial cells. The proportion of rumen epithelial cells in the G_0_/G_1_ phase in the NM group was significantly lower than that in the NC group (*P* < 0.05), whereas the proportion of cells in the S phase was higher (*P* < 0.05). These results indicate that the addition of yeast culture to the diet induces rumen epithelial cells to undergo accelerated cell cycle progression, which is characterized by a shorter duration of the G_0_/G_1_ phase. Using PCNA allows for differentiating resting/quiescent cells from proliferating cells. The present study revealed a significant increase in PCNA-positive cells in the ruminal epithelium of lambs fed the yeast-containing diet. These findings further show that yeast culture promotes rumen development, which is associated with accelerated cell cycle progression. These changes ultimately increase rumen papillae, enhancing nutrient absorption.

Cell cycle protein-dependent kinases (CDKs) and their regulatory cell cycle protein subunits are pivotal in regulating cell cycle progression [58]. To gain deeper insights into the molecular mechanisms through which yeast culture promotes the proliferation of rumen epithelial cells, we investigated the mRNA and protein expression of rumen epithelial cell cycle proteins using quantitative reverse transcription (qRT)-PCR and western blot. CDKs, such as CDK4/6, CDK2, and CDK1, are serine/threonine kinases with various substrates. CDK4/6 complexes with D-type cyclins activate and phosphorylate retinoblastoma protein (Rb) in the early G_1_ phase, releasing the transcription factor E2F [57, 59–62], which in turn promotes the transcription of early E2F responsive genes, including the A- and E-type cyclins [57, 63]. In the late G_1_ phase, cyclin E activates CDK2, resulting in complete Rb phosphorylation and E2F-driven transcription [57, 60]. These events push cells through the G_1_/S restriction point, starting the S phase [64, 65]. During the S phase, A-type cyclins bind to CDK2 to phosphorylate DNA replication proteins, advancing the cell to the G_2_ phase [66]. In late G_2_, CDK1/cyclin A activates the G_2_/M transition and initiates prophase [67]. Finally, CDK1/cyclin B complexes are formed in the M phase, completing mitosis. The findings of this study demonstrated that the administration of yeast culture as a feed additive increased the expression of genes encoding *CDKs 4*, *6*, and *2*, as well as cyclins D1, E, A, and B in rumen epithelial cells of lambs. Similar changes were observed in the expression levels of Cdk2, Cdk4, Cdk6, cyclin D1, and cyclin E1. These results indicated that rumen epithelial cells of lambs supplemented with yeast culture exhibited accelerated cell cycle progression. The findings of this study are consistent with those of previous studies showing that an increase in fatty acids produced in the rumen is associated with a rapid increase in the mitotic index of rumen epithelial cells [38, 67–70].

Similarly, apoptosis plays a significant role in the development of rumen papillae [25, 38, 71–73]. Apoptosis, a highly regulated process of cell death, is a normal physiological process common to multicellular organisms. In the initial stages of apoptosis, cells undergo karyotyping. The present study revealed that the number of TUNEL-positive cells in the rumen epithelium of lambs supplemented with yeast culture was significantly lower than that of the control group.

Nuclear alterations and mitochondrial enlargement in the subcellular structures of the rumen epithelium of control lambs were examined using TEM. The findings indicate that the apoptosis rate of rumen epithelial cells was diminished in lambs administered yeast culture. Two principal pathways of apoptosis have been identified: extrinsic and intrinsic. The extrinsic pathway is defined as the receptor-mediated initiation of apoptosis. Apoptosis-associated membrane receptors are members of the tumor necrosis factor-α (TNF-α) receptor superfamily, whose activation depends on two major ligands: The interaction between tumor necrosis factor (TNF) and its receptor transmits death signals through the recruitment of the tumor necrosis factor receptor-associated death domain (TRADD) and the Fas-associated death domain proteins (FADDs), affecting programmed cell death through the action of cysteine [74–76]. The effect of ASNase on programmed cell death has been heavily investigated. Procaspase 8 undergoes autocleavage to produce caspase 8, which initiates the execution phase of cell death. Caspase-8 cleaves procaspase 3 through protein hydrolysis to produce caspase 3, which is responsible for the eventual execution of protein hydrolytic degradation of various intracellular proteins [77, 78]. The present study revealed a significant reduction in *caspase 3*, *caspase 8*, *Fas*, and *TNFR1* expressions in rumen epithelial cells of lambs supplemented with yeast culture. These findings indicate that the administration of yeast culture through the supplemental feeding route can effectively mitigate receptor-mediated apoptosis in the rumen epithelial cells of lambs. The intrinsic apoptotic pathway is regulated by mitochondria.

The presence of a stimulus disrupts the mitochondrial transmembrane potential, leading to the dispersion of the membrane potential and increased membrane permeability [79–82]. Stimulation also results in the formation of a mitochondrial permeability transition pore (MPT) within the outer membrane, releasing proapoptotic factors into the cytosol [67]. The apoptotic bodies cleave procaspase 9 to produce active caspase 9, activating effector caspase 3. In the present study, we observed a significant decrease in *Cyt-c*, *Bax*, and *Bad* expressions in the rumen epithelial cells of lambs supplemented with yeast culture. Cyt-c, Bax, C-caspase 3, and C-caspase 7 protein expressions were also significantly diminished in the rumen epithelial cells of lambs supplemented with yeast culture. These findings indicate that providing yeast culture as a feed additive can effectively inhibit the intrinsic apoptotic signaling pathway in the rumen epithelial cells of lambs.

Growth-promoting factors, such as IGF, fibroblast growth factor (FGF), and epidermal growth factor (EGF), can promote the proliferation of rumen epithelial cells in vitro [83]. These genes may regulate the proliferation and morphogenesis of rumen epithelial cells in vivo. In this study, there was no evidence for FGF differential expression in the protein data; however, fibroblast growth factor binding protein 1 (FGFBP1) was highly expressed in the rumen epithelial cells of lambs supplemented with yeast culture (Additional File 2). Most EGF is produced in the parotid gland and transported to the rumen through saliva; only minimal expression is observed in the rumen tissue [83]. The potential roles of FGF and EGF in promoting rumen epithelial cell proliferation were not the primary focus of this study.

Previous studies have demonstrated a positive correlation between plasma IGF-1 concentration and rumen papilla proliferation [38, 84–86]. The IGF family has attracted considerable research interest, with studies demonstrating a correlation between IGF-1 plasma concentrations and rumen papillary growth in ruminants fed high levels of butyrate [87] and energy [88]. The present study observed an elevation in plasma IGF-1 levels in lambs supplemented with yeast culture for one month. IGF-1 exerts growth-promoting effects by binding to IGF-1R, a tyrosine kinase receptor that triggers a cellular signaling cascade that promotes tissue growth upon activation. Furthermore, promotion of rumen epithelial cell proliferation by IGF-1 is regulated by IGF-binding proteins (IGFBPs). IGFBP 2, 3, 5, and 6 regulate the proliferative effects of IGF-1 on rumen epithelial cells [89, 90]. IGFBP 5 enhances the function of IGF-1, specifically promoting rumen epithelial cell proliferation when upregulated [91–93]. Notably, IGFBP 3 can potentially inhibit the action of IGF-1. Consequently, a reduction in the expression of this protein may facilitate the proliferation of ruminal epithelial cells. Data from the present study demonstrate that *IGFBP 2*, *5*, and *6* mRNA expressions were upregulated in the rumen epithelial cells of lambs supplemented with yeast culture. However, no significant difference was observed in *IGFBP3* mRNA expression compared with that in the control group. Considering these findings, it can be postulated that alterations in the SCFA concentration in the rumen epithelial cells of lambs supplemented with yeast culture may regulate IGFBP expressions, influencing rumen epithelial cell proliferation and differentiation. This study provides deeper insights into the molecular mechanisms underlying the promotion of rumen papilla development in lambs following administration of yeast culture.

This study has some limitations. First, the experimental design of the present study was primarily in vivo; therefore, validation studies using cellular models remain warranted. Isolated and cultured rumen epithelial cells can be used to investigate the direct effects of various biomolecules from different internal rumen environments on rumen epithelial cells. The molecular mechanisms underlying rumen papilla development are influenced by several factors, including the epithelial barrier, rumen microbial ecology, metabolism, cell proliferation, and apoptosis. However, due to the limitations in the experimental conditions, we could only investigate the effects of a few key factors on the cells; therefore, the potential effects of other factors on rumen papilla development warrant further investigations. Future studies are required to fully elucidate the molecular mechanisms involved in rumen papilla development and validate our results.

## Conclusions

**Fig. 14:**
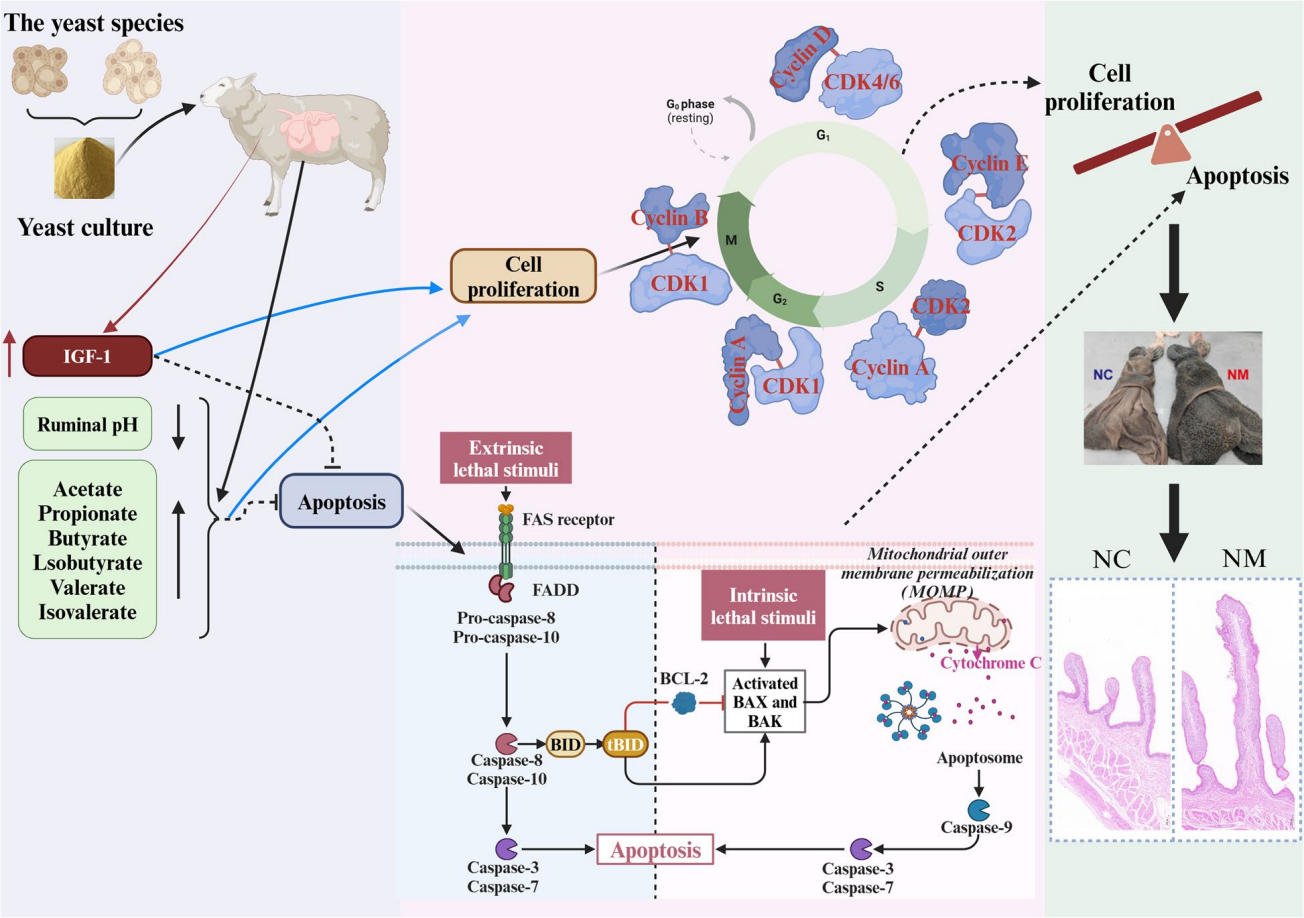
Schematic representation of the mechanism through which composite yeast culture promotes rumen epithelial growth in lambs by modulating rumen epithelial cell cycle and apoptosis

This study demonstrated that the provision of yeast culture to lambs can enhance their production performance, stimulate the proliferation of cells associated with the rumen epithelium, inhibit apoptosis, and upregulate the expression of genes involved in VFA uptake and metabolism in the rumen epithelium of lambs.Based on the results of this study, we propose a molecular mechanism by which feeding with yeast culture promotes the development of the rumen epithelium in lambs (Fig. 14): supplemental yeast culture feeding improves the growth performance of lambs, enhances rumen fermentation capacity, and promotes rumen epithelial development. Notably, supplementation with yeast culture elevates the concentration of IGF-1 in the plasma of lambs. Furthermore, yeast culture increased the amount of butyrate in the rumen. Butyrate in the rumen can directly stimulate the development of rumen papillae. Butyrate accelerates the cell cycle and inhibits the onset of apoptosis. An increase in plasma IGF-1 concentration and rumen epithelial IGF-1R expression is another contributing factor to the promotion of rumen epithelial development in lambs through supplemental yeast culture feeding. Due to these factors, there is a significant elevation in CDK and associated regulatory cell cycle protein expressions, accompanied by enhanced progression of the cell cycle in rumen epithelial cells of lambs supplemented with yeast culture. Concurrently, the expression of proteins associated with the extrinsic and intrinsic apoptotic pathways and the apoptosis rate were diminished. Ultimately, the rumen papillae of lambs supplemented with yeast culture were enlarged, which enhanced nutrient uptake and improved production performance. To the best of our knowledge, this is the first report on the effects of supplemental yeast culture feeding on rumen epithelium-associated cell proliferation, apoptosis, and VFA uptake and metabolism protein and mRNA expressions. These findings provide novel insights into the molecular mechanisms through which yeast culture promotes the development of rumen epithelium in weaned lambs, which will aid in the development of precise feeding techniques to enhance the productivity of feedlot-weaned lambs.

## Supplementary Information

Additional file 1: Downregulation of DEPs.

Additional file 2: Upregulation of DEPs.

Additional file 3: Enrichment of DEPs according to GO.

Additional file 4: Enrichment of DEPs based on COG.

Additional file 5: KEGG pathway enrichment of DEPs.

Additional file 6: Classification of proteins according to differences in expression ploidy.

Additional file 7: KEGG pathway clustering analysis.

Additional file 8: KEGG cell cycle pathway.

Additional file 9: KEGG apoptosis pathway.

Additional file 10: DEPs interaction analysis.

## Data Availability

The datasets used and analyzed during this study are available from the corresponding author upon reasonable request.

## Abbreviations

APOB: Apolipoprotein B
BSA: Bovine serum albumin
CDKs: Cell cycle protein-dependent kinases
Cyto-c: Cytochrome C
DMI: Dry matter intake
DEP: Differentially expressed proteins
EGF: Epidermal growth factor
FDR: False-positive rate
FGF: Fibroblast growth factor
FGFBP1: Fibroblast growth factor binding protein 1
GO: Gene Ontology
H&E: Hematoxylin and eosin
HDDC: 25′-Deoxynucleotidase
IGF-1: Insulin-like growth factor 1
IGF-1R: IGF-1 receptor
IGFBPs: IGF-binding proteins
KEGG: Kyoto Encyclopedia of Genes and Genomes
NDUFB10: NADH dehydrogenase one beta subcomplex subunit 10
PCA: Principal component analysis
PPI: Protein-protein interaction
PBS: Phosphate-buffered saline
PVDF: Polyvinylidene fluoride
PRSS42P: Serine protease 42
PFDN1: Prefoldin subunit 1
PCNA: Proliferating cell nuclear antigen
qRT-PCR: Quantitative reverse transcription polymerase chain reaction
RSD: Relative standard deviation
SLC9A1: Sodium/hydrogen exchanger
TPM1: Tropomyosin 1
TUNEL: Terminal deoxynucleotidyl transferase dUTP nick-end labeling
TEM: Transmission electron microscopy
TMEM168: Transmembrane protein 168
VFA: Volatile fatty acid
