# Supplementary figures and images for: Yeast culture in weaned lamb feed: a proteomic journey into enhanced rumen health and growth
Source: J Anim Sci Biotechnol. 2025 Aug 1;16:107. doi: 10.1186/s40104-025-01223-8 (PMC12315397; doi:10.1186/s40104-025-01223-8)

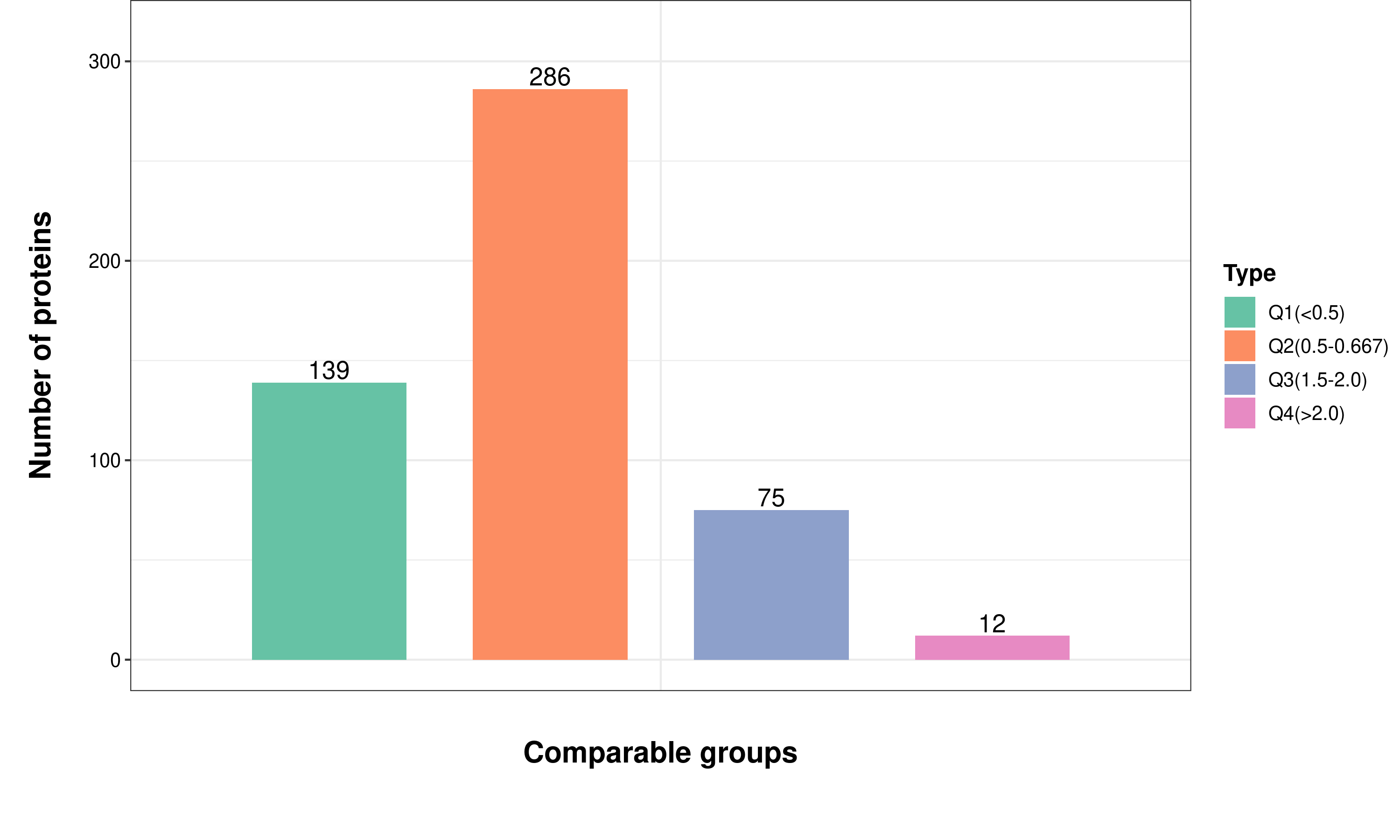

Supplement: Supplementary file 6 — Additional file 6: Classification of proteins according to differences in expression ploidy. [file 40104_2025_1223_MOESM6_ESM.png]

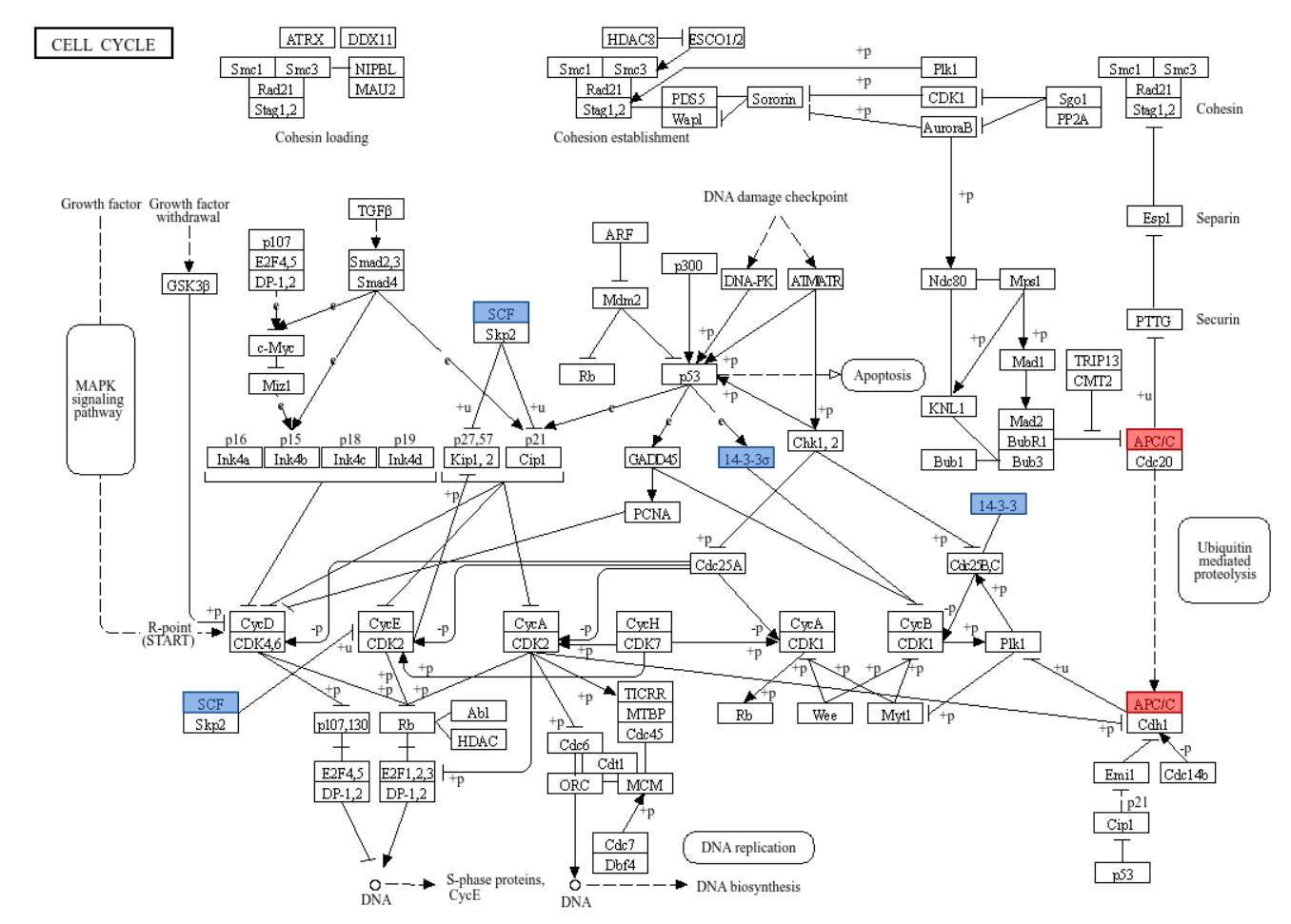

Supplement: Supplementary file 8 — Additional file 8: KEGG cell cycle pathway. [file 40104_2025_1223_MOESM8_ESM.png]

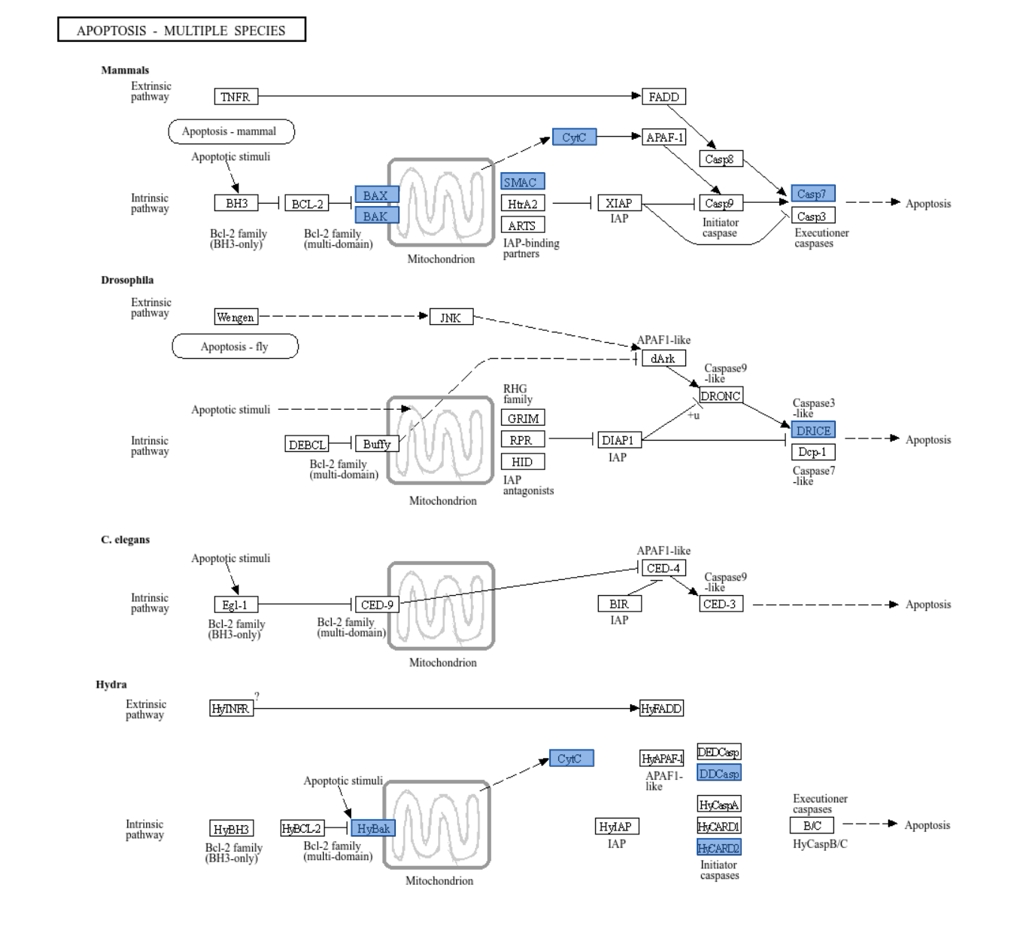

Supplement: Supplementary file 9 — Additional file 9: KEGG apoptosis pathway. [file 40104_2025_1223_MOESM9_ESM.png]
